# Chemical Engineering of Altermagnetism in Two-Dimensional Metal–Organic Frameworks

**DOI:** 10.1021/jacs.5c22589

**Published:** 2026-05-14

**Authors:** Diego López-Alcalá, Alberto M. Ruiz, Andrei Shumilin, José J. Baldoví

**Affiliations:** Instituto de Ciencia Molecular, 16781 Universitat de València, Catedrático José Beltrán 2, 46980 Paterna, Spain

## Abstract

Altermagnetism represents a novel magnetic phase exhibiting nonrelativistic spin splitting without net magnetization driven by lattice symmetry. Here, we introduce a general coordination-chemistry-based strategy to realize and control altermagnetic (AM) spin splitting in two-dimensional (2D) planar tetracoordinated Cr-based metal–organic frameworks (MOFs). Using density functional theory (DFT) calculations, we demonstrate that ligand symmetry and its arrangement within the lattice can be used to lower the crystallographic symmetry of Cr-based MOFs, enabling g-wave AM spin splitting up to 65 meV. Furthermore, frontier molecular orbital engineering (FMOE) allows selective ligand spin polarization, inducing a shift to d-wave AM anisotropy in polycyclic ligand-based 2D MOFs with spin splitting up to 83.9 meV. Microscopic magnetic exchange interaction (*J*) analysis reveals that ligand-mediated interactions dominate over metal–metal coupling, stabilizing AM order in systems with spin-polarized ligands. Interestingly, we further confirm AM spin splitting in the spin wave spectrum, where chiral magnon splitting is observed. Finally, we show that AM spin splitting gives rise to experimentally accessible charge-to-spin conversion, emerging as a linear response in d-wave and as a symmetry-allowed nonlinear effect in g-wave 2D AM MOFs. This work establishes coordination chemistry as a powerful and versatile route to symmetry control in 2D MOFs, enabling the rational design of 2D molecular materials with tunable electronic and AM properties for next-generation spintronic devices.

## Graphical abstract

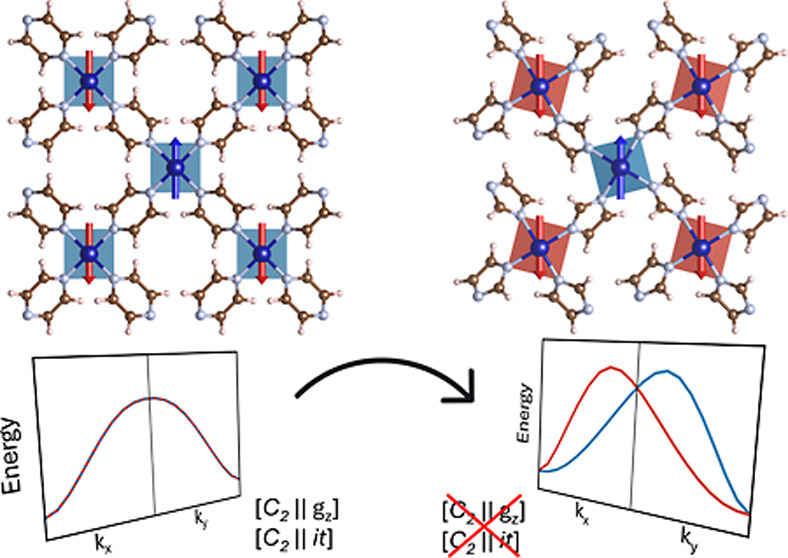

## Introduction

Spintronics has grown rapidly as controlling the spin degree of freedom enables key functionalities such as giant magnetoresistance, spin-transfer torque, and electrically driven spin–orbit coupling (SOC) effects.^1–3^ These rely on magnetic interactions and spin alignment, classically described by ferromagnetic (FM) or antiferromagnetic (AFM) order. Beyond these conventional configurations, a variety of unconventional magnetic states have been identified, including noncollinear textures and topological spin configurations.^4,5^ Among them, altermagnetism stands out as a new magnetic phase that displays sizable nonrelativistic spin splitting despite zero net magnetization.^6–8^ This behavior originates from the lattice symmetry: crystallographic rotation operations relate opposite spin sublattices in a way that cannot be reproduced by inversion (*i*), translation (*t*), or their combinations.^9^ Consequently, the degeneracy between opposite spin channels is lifted in momentum space while the real-space magnetic structure remains strictly AFM. This unique combination of AFM order and momentum-space spin splitting enables technologically relevant functionalities, including highly anisotropic spin currents, efficient spin filtering without SOC, and electrically controllable spin transport responses.^10^ In this context, several strategies have been developed to engineer the symmetry conditions required for altermagnetism, including the application of external electric fields, Janus architectures, and twistronics.^11^ Experimentally, room-temperature altermagnetic (AM) spin splitting has been confirmed both in the electronic structure by angle-resolved photoemission spectroscopy (ARPES)^12,13^ and in the spin-wave spectrum through inelastic neutron scattering (INS) measurements.^14^

Metal–organic frameworks (MOFs) are periodic architectures in which metal centers are connected through organic ligands.^15–17^ Their high degree of chemical tunability, rooted in the versatility of the ligands, has positioned MOFs as platforms for unprecedented functionalities across diverse fields, leading to their recognition with the Nobel Prize in Chemistry in 2025.^18^ In particular, magnetic MOFs have recently emerged as promising candidates for spintronic devices.^19–22^ Taking advantage of coordination chemistry, molecular solids offer a versatile route to engineer coupled magnetic, electric, and orbital degrees of freedom.^23–25^ Remarkably, Pedersen et al. demonstrated that incorporating open-shell organic linkers enables strong metal–radical magnetic interactions in layered CrCl_2_(pyz)_2_ (pyz = pyrazine),^26^ where postsynthetic reduction yields robust ferrimagnetism with Curie temperatures up to 515 K and large coercivity.^27,28^ Beyond bulk crystals, exfoliation down to the monolayer limit has been achieved in related pyz-based layered MOFs^29,30^ and theoretically predicted to be experimentally feasible in CrCl_2_(pyz)_2_.^31,32^ Building on the remarkable properties of pyz-based systems, a rapidly expanding landscape of structural and electronic modifications has been proposed to engineer new functionalities. Tunable electronic and magnetic order has been achieved through valence tautomerism,^33–35^ metal substitution,^36^ and applied pressure,^37^ while additional phenomena such as multiferroicity^38,39^ and 1D Cr–pyz motifs^40^ have recently been realized. Beyond pyz linkers, planar tetracoordinated Cr-based MOFs have also been predicted to host novel functionalities and enhanced magnetic behavior through frontier molecular orbital engineering (FMOE).^41–44^

In this context, magnetic MOFs emerge as a promising platform for realizing AM lattices since the chemical versatility of coordination networks enables the design of structures with tailored symmetrical properties. These systems have also been highlighted as platforms for magneto-optical responses, such as Kerr effects, driven by symmetry and magnetic order without conventional ferromagnetism.^45^ Theoretical investigations of 2D MOF candidates for altermagnetism published to date have typically relied on symmetry breaking via selective spin polarization on the ligands,^46,47^ employing bilayer stacking,^48^ or constructing mathematical lattice architectures.^49^ Recent theoretical studies have further proposed AM MOFs in the context of multiferroicity^50,51^ and Lifshitz transitions.^52^ However, symmetry breaking driven directly by coordination chemistry remains largely unexplored as a systematic route in the literature, representing a promising route for designing 2D AM-MOFs with tunable properties. Taking advantage of the vast library of organic linkers that fulfill the symmetry requirements for AM spin splitting, a large number of candidate MOFs can be engineered with tailored electronic and magnetic properties.

In this work, we demonstrate that ligand symmetry provides a powerful route to tailor alternating magnetic spin splitting in 2D MOFs. We first identify clear evidence of AM spin splitting in a 2D MOF by modifying the symmetry of the coordination network through the choice and arrangement of the organic linkers, which lower the lattice symmetry and enable g-wave AM spin splitting. Furthermore, we employ FMOE to selectively induce spin polarization on the ligand scaffold in related 2D MOFs with a reduced lattice symmetry. Remarkably, the sublattice symmetry breaking driven by ligand spin polarization produces a transition to d-wave AM anisotropy. Moreover, varying the degree of conjugation within the organic linkers enables systematic transitions from insulating to narrow-band gap semiconducting states. Finally, we address the spin-dependent transport fingerprints of the AM spin splitting. While d-wave altermagnets exhibit a spin-dependent conductivity, symmetry forbids such a response in g-wave systems, where spin splitting instead emerges as a nonlinear effect at the third order in the applied electric field. We show that both regimes give rise to experimentally accessible charge-to-spin conversion in 2D AM MOFs. Together, these results establish a general strategy for the rational design of altermagnetism in 2D MOFs, enabling coordination chemistry to tune electronic and magnetic properties in molecular materials for spintronic applications.

## Results and Discussion

### Ligand-Symmetry-Driven Design Strategy and Structures of 2D AM MOFs

As illustrated in Figure 1a, the AFM coupling between metal centers in a 2D MOF lattice coordinated by pyz ligands (M­(pyz)_2_) gives rise to a conventional AFM electronic band structure when the spin polarization resides on the metal centers. This behavior arises from the high crystallographic symmetry of the lattice. The *P*4/*nbm* space group contains several symmetry operations that relate the two magnetic sublattices, including the *g*_*z*_ glide symmetry with a reflection plane at *z* = 0 as well as a combined inversion and translation operation (*it*). When combined with a *C*_2_ rotation in spin space, these operations are conventionally denoted as [*C*_2_||*g*_*z*_] and [*C*_2_||*it*].^6^ When the real space component (*S*) of a combined symmetry [*C*_2_||*S*] conserves or inverts the electron momentum, as is the case for both *g*_*z*_ and *it* in the 2D M­(pyz)_2_ lattice, the electronic bands are symmetry-constrained to remain spin-degenerate, resulting in conventional antiferromagnetism. The high symmetry of M­(pyz)_2_ ultimately originates from the *D*_2h_ point group of the pyz ligand, which preserves the combined symmetries [*C*_2_∥*g*_*z*_] and [*C*_2_∥*it*] throughout the lattice.

**Figure 1:**
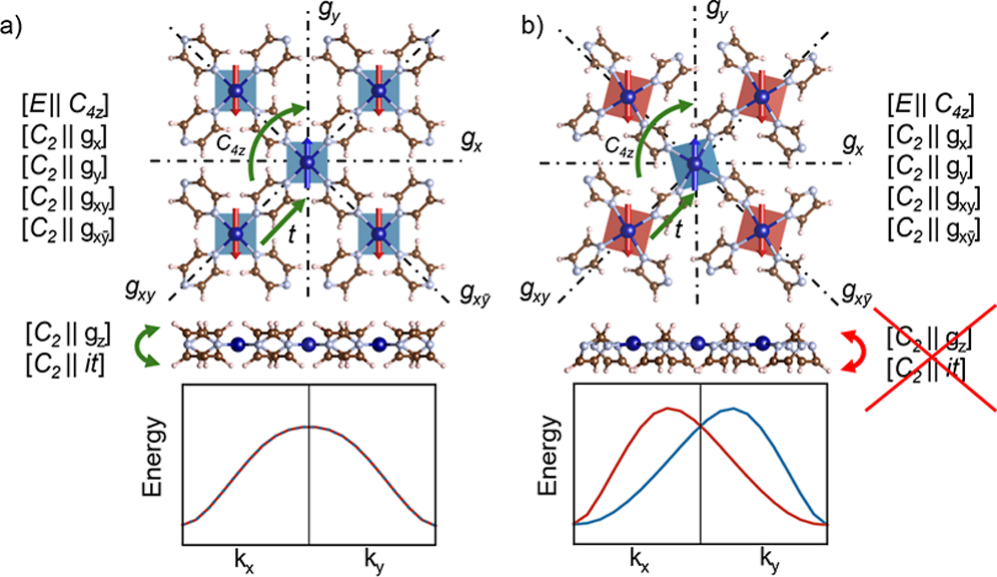
(a) Representative pyz-based 2D MOF antiferromagnetically coupled illustrating all the possible symmetry operations that connect spin sublattices and gives rise to conventional AFM spin degeneracy. (b) Representative imz-based 2D MOF antiferromagnetically coupled, where ligand-symmetry breaks the combined spin–lattice symmetries enforcing spin degeneracy, enabling AM spin splitting. Color code: blue (metal), brown (C), cyan (N), and white (H).

In this context, a viable strategy to induce controlled symmetry breaking in 2D MOF lattices based on M­(pyz)_2_ requires the rational chemical design of a lattice with a noncentrosymmetric analogue of the pyz ligand. Imidazole-based (imz) linkers emerge as an ideal candidate for this purpose, as its molecular structure lacks an inversion center due to its *C*_2v_ point-group symmetry.^53^ Figure 1b illustrates a 2D M­(imz)_2_ lattice in which the metal centers remain AFM-coupled, in close analogy to the pyz-based structure. However, the absence of inversion symmetry in the imz linker breaks the combined symmetries [*C*_2_||*g*_*z*_] and [*C*_2_||*it*], thereby lifting the spin degeneracy and allowing spin splitting of the electronic bands. The resulting *P*4*bm* space group nevertheless retains several glide symmetries, namely, [*C*_2_||*g*_*x*_], [*C*_2_||*g*_*y*_], [*C*_2_||*g*_*xy*_], and $\left[C_{2}∥g_{x\mathrm{y̅}}\right]$ , with reflection planes at *x* = 0, *y* = 0, *x* = *y*, and *x* = −*y*, respectively (see Supporting Information Section 5). It also contains the combined symmetry [*E*||*C*_4z_], where *E* denotes the unitary identity operation in spin space and *C*_4z_ corresponds to a 90° rotation about the *z* axis in real space. Because glide symmetry operations neither conserve nor invert electron momentum, they do not enforce conventional antiferromagnetism. At the same time, they preserve the equivalence between the two magnetic sublattices, thereby excluding ferrimagnetism and resulting in an AM electronic band structure.

Consequently, we perform density functional theory (DFT) calculations in a 2D Cr­(imz)_2_ lattice to study the origin of symmetry breaking and its implication on the origin of altermagnetism in this 2D MOF structure. We consider Cr atoms as a metal center for the formation of the Cr­(imz)_2_ lattice since it has been extensively studied in related pyz-analogue MOFs. The resulting structure belongs to the *P*4*bm* space group with lattice parameters *a* = *b* = 8.76 Å. Noncentrosymmetric imz ligand generates alternating patterns of clockwise and counterclockwise orientation of the ligands around the metal centers (Figure 1b). A small deviation of ∼5° from planarity in square-planar coordinated Cr atoms is observed due to the ligand environment and the tilting of the imz rings is 47.7° from the 2D plane. An alternative ligand-orientation pattern lies 0.36 eV per Cr atom above the *P*4*bm* structure in energy (Figure S3). To accurately describe the electronic structure of 2D MOFs, we employ the hybrid HSE06 functional. Figure 2a shows the electronic band structure and projected density of states (PDOS) of Cr­(imz)_2_, where an indirect band gap (X → Γ) of 4.8 eV is deduced. An absence of spin splitting along the Γ–X–M−Γ high-symmetry path is observed, whereas along the X–Y path, one can notice that different spin component bands are nondegenerated. Equally occupied states of spin up and down in PDOS can confirm the altermagnetism in Cr­(imz)_2_ since there is no net magnetization along the lattice, whereas a noticeable spin splitting is present at the band structure. Figure 2b shows the AM splitting at both valence (VB) and conduction bands (CB), which increases up to 43.8 and 65.1 meV in the region close to the Fermi level in the VB and CB, respectively. Tables S2 and S3 compare the results obtained using different exchange–correlation functionals and DFT parameters. The consistency of these results demonstrates that the predicted intrinsic altermagnetism in Cr­(imz)_2_ is robust and does not arise as an artifact of the computational framework. A complete picture of AM splitting along the 2D MOF plane is depicted in Figure 2c. Following the definition of the characteristic spin-group integer,^6^ 2D g-wave-like anisotropy is identified from the presence of four spin-degenerate nodal planes crossing the Γ point (*kx* = 0, *ky* = 0, and *kx* = ±*ky*) in the Brillouin zone. Those nodes in the AM wave correspond to the Γ–X −M−Γ high-symmetry path, whereas in the X–Y path, an alternated spin splitting is observed. Additionally, we compute the band structure of Cr­(imz)_2_ including SOC and find no noticeable changes in the electronic bands (Figure S7), thereby confirming the nonrelativistic origin of the AM spin splitting.

**Figure 2:**
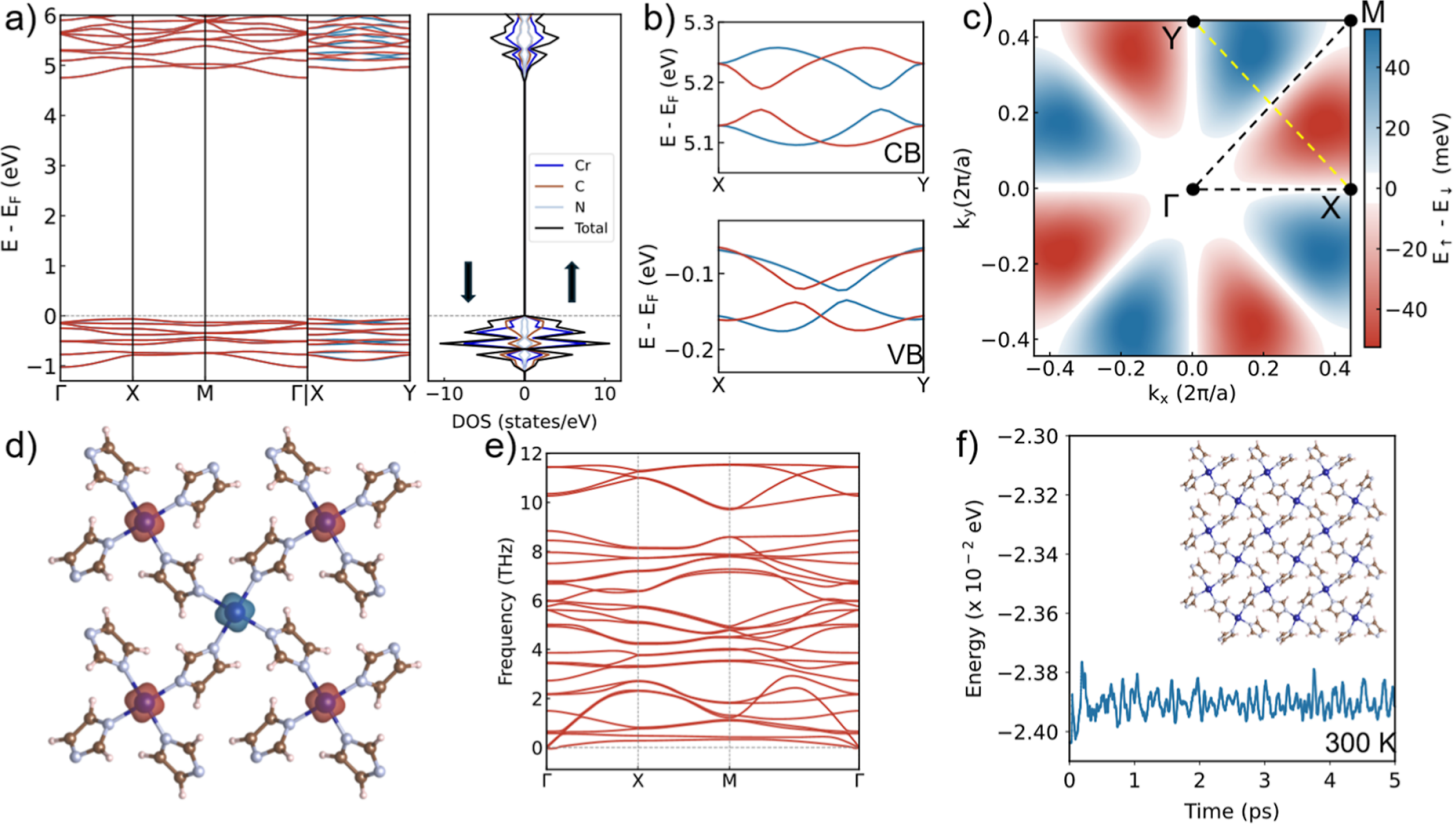
(a) Band structure and PDOS of Cr­(imz)_2_ calculated using the hybrid HSE06 functional. (b) Zoom of CB and VB regions along the high-symmetry path with AM spin splitting. (c) Spin splitting in VBM along the 2D MOF plane. (d) Spin density in Cr­(imz)_2_. The blue (red) isosurface represents the spin-up (down) component. (e) Phonon spectrum and (f) AIMD simulation at 300 K in Cr­(imz)_2_.

Altermagnetism is allowed in Cr­(imz)_2_ since AFM coupling between metal centers is observed, the FM configuration being higher in energy by 8.4 meV/Cr atom. In this system, spin density is restricted to the Cr atoms since the ligands do not show significant spin polarization (Figure 2d). Calculated magnetic moments are 3.7 μ_B_ in Cr atoms, compatible with reported Cr­(II)-based planar tetracoordinated MOFs in the literature.^28^ We conduct phonon analysis and *ab initio* molecular dynamics (AIMD) simulations to elucidate the structural and thermodynamic stability of the Cr­(imz)_2_ 2D MOF. Figure 2e shows the calculated phonon dispersion of the structure. Minimal imaginary frequencies appear near the Γ point, which are commonly observed in MOFs due to the presence of low-frequency collective modes and the sensitivity of phonon calculations in large, flexible frameworks, due to numerical limitations such as finite supercell sizes, convergence thresholds, and the high density of vibrational modes characteristic of these systems.^54–58^ Indeed, AIMD simulations show that the 2D MOF structure is preserved at room temperature (Figure 2f), supporting its dynamical stability. At temperatures up to 600 K, ligand rotations are thermally activated (Figure S2), with no evidence of structural collapse or phase transition.

### Frontier Molecular Orbital Engineering of 2D AM MOFs

The conclusions extracted from above can be extrapolated to many 2D MOFs which fulfill the symmetry criteria for altermagnetism, due to the chemical versatility of organic linkers. Therefore, we extend the electronic and magnetic properties study to the 2D MOFs depicted in Figure 3. We construct a set of Cr planar tetracoordinated lattices with noncentrosymmetric ligands analogous to imz, containing both monocyclic and polycyclic organic linkers. Analogous to Cr­(imz)_2_, a symmetry breaking between adjacent Cr sublattices is observed in all structures. A symmetry analysis of the fully relaxed geometries confirms that the *P*4*bm* lattice symmetry is preserved within numerical tolerance, with small deviations arising from the internal degrees of freedom of the organic rings rather than from a cooperative structural distortion. For the polycyclic-ligand MOFs, the clockwise ligand orientation remains energetically favored, lying 0.54 eV per Cr atom lower in energy than the alternating ligand-orientation pattern (Figure S21). Changes in ligand coordination not only alter the distance between magnetic Cr atoms in the lattice but also modify the electronic and magnetic interactions in the 2D MOFs. Importantly, different ligand conjugations enable distinct effective electronic states of the organic linkers within the coordination network. In particular, while some ligands behave as electronically innocent, remaining closed-shell upon coordination, others exhibit a redox noninnocent character that allows partial metal–ligand charge redistribution and the emergence of ligand-centered spin density. We compute phonon dispersions and AIMD simulations for Cr­(tdz)_2_, Cr­(DApent)_2_, and Cr­(DAind)_2_ (see Supporting Information Sections 2–4), and in all cases, we observe thermodynamic stability, as no indications of phase transitions arise during the AIMD trajectories. The phonon dispersions of the polycyclic-ligand MOFs, e.g., Cr­(DApent)_2_ and Cr­(DAind)_2_, display small negative frequencies (Figures S20 and S30), as also observed for Cr­(imz)_2_. As discussed above, this is a well-known feature of harmonic phonon calculations in flexible MOF systems. Consistently, AIMD simulations show no structural distortions, confirming the stability of the optimized geometries.

**Figure 3:**
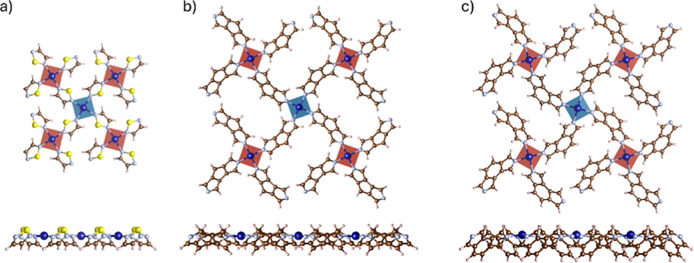
Top and side views of (a) Cr­(tdz)_2_, (b) Cr­(DApent)_2_, and (c) Cr­(DAind)_2_. Color code: blue (metal), brown (C), cyan (N), yellow (S), and white (H).

Interestingly, we find that the spin distribution along the lattice is closely linked to the energy alignment of the frontier molecular orbitals in the MOFs. In particular, the energy separation between the metal-centered occupied states and the ligand LUMO (Δ_*CT*_) provides direct insight into the ability of the organic linkers to participate in the magnetic structure upon coordination. Large Δ_*CT*_ values favor an electronically innocent, closed-shell ligand configuration, leading to spin localization at the Cr centers. In contrast, reduced Δ_*CT*_ values make ligand-based frontier orbitals energetically accessible, enabling partial metal–ligand charge redistribution and the emergence of a redox noninnocent, radical-like ligand state that can host exchange-induced spin polarization. Importantly, this process preserves the high-spin *d* ^4^ configuration of Cr­(II), while introducing ligand-centered magnetic moments that strongly reshape the magnetic exchange network. Similar FMOE-driven electronic rearrangements, often described as tautomeric or valence-adaptive behavior, have been reported in related magnetic MOFs and are known to induce distinct magnetic ground states.^33–35,59^ Additionally, we conduct cluster DFT calculations on the isolated ligands to investigate their HOMO–LUMO gaps (Δ_*L*_) (see Supporting Information Section 6), which are indicative of the ability of the organic linker to accommodate spin polarization in the coordination network. These calculations reveal that ligands displaying distinct electronic responses are associated with different effective frontier orbital configurations, reflecting differences in their ability to accommodate ligand-centered spin density. In particular, ligands that develop spin polarization in the extended framework are characterized by an increased redox activity at the ligand level, without altering the magnetic configuration of the metal centers and within an overall charge-balanced coordination network. Despite both methods being conceptually different, we observed a noticeable alignment of their results, which enforces the conclusions extracted from the analysis of the impact of Δ_*CT*_ on the distribution of spin polarization. Figures 4a and b show the calculated Δ_*CT*_ and Δ_*L*_ values for each organic linker via periodic and cluster DFT, respectively. In the case of monocyclic linkers (Figure 4a), one can see that imz presents larger gaps than tdz. This aligns perfectly with the observation of confined spin polarization at the metal in Cr­(imz)_2_ and the spin-polarized ligands in Cr­(tdz)_2_ (Figures S12 and S13). On the other hand, polycyclic organic linkers (Figure 4b) show the same trend for DApent and DAind, where the former shows larger gaps compared to the latter. Therefore, spin density is localized at the metal centers in Cr­(DApent)_2_ (Figure S22), whereas spin polarization is observed over the ligand scaffold in Cr­(DAind)_2_ (Figure 4c). Bader charge analysis performed for all the considered 2D MOFs reveals very similar net charges of −0.8 *e* on each ligand and +1.6 *e* on the Cr centers across the different coordination networks, indicating the absence of significant ligand-dependent charge transfer (Table S22).

**Figure 4:**
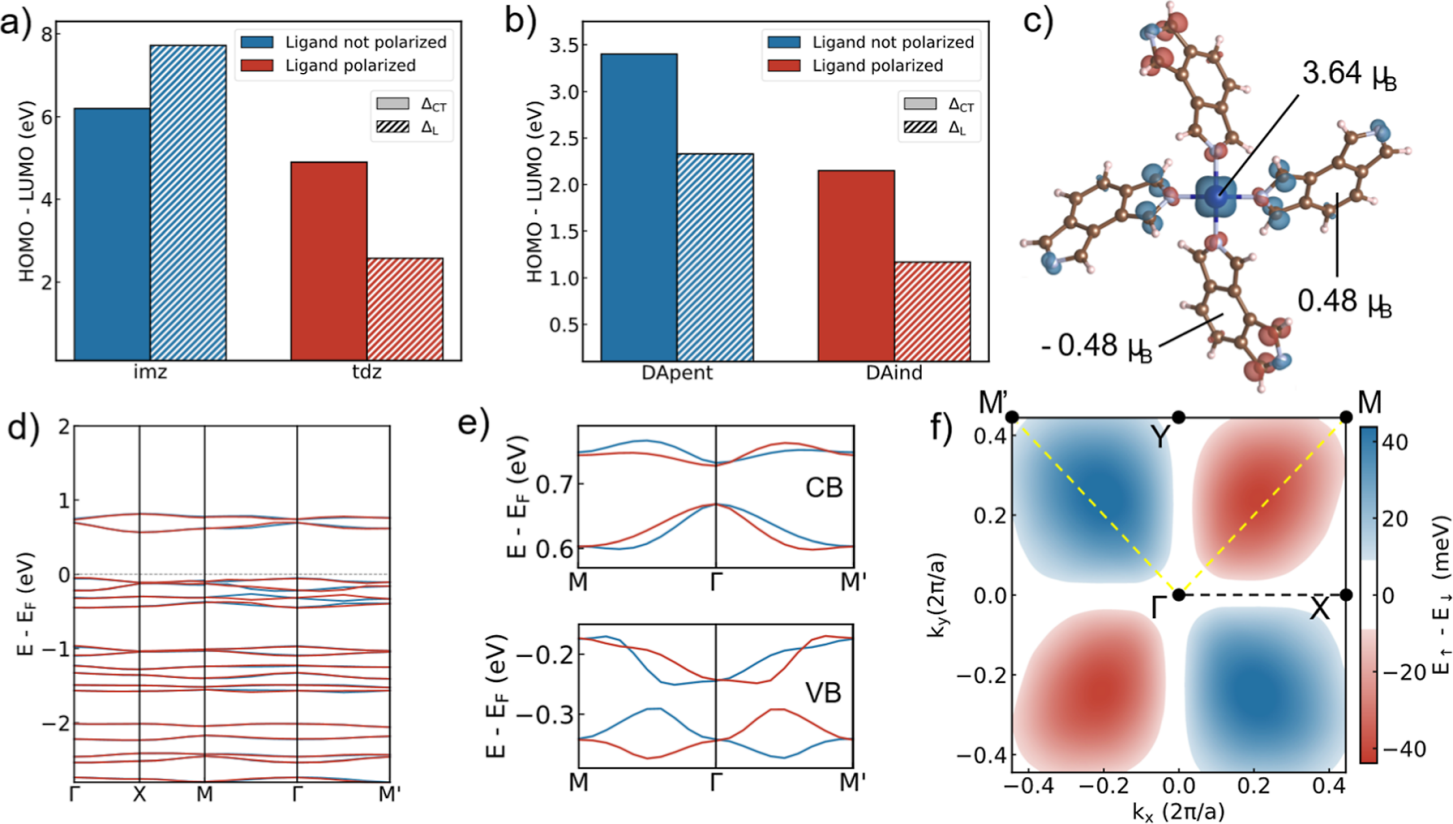
Calculated Δ_*CT*_ and Δ_*L*_ for (a) monocyclic- and (b) polycyclic-based 2D MOFs. Blue (red) color represents spin-unpolarized (polarized) ligands. (c) Spin density with magnetic moments, (d) band structure calculated using the hybrid HSE06 functional, (e) zoom of CB and VB regions along the high-symmetry path with AM spin splitting, and (f) spin splitting in VBM along the 2D MOF plane in Cr­(DAind)_2_.

Redistribution of spin polarization along the lattice has an enormous impact on the electronic structure of 2D MOFs. Figure 4d shows the band structure for AM Cr­(DAind)_2_, where a direct band gap (Γ → Γ) of 0.61 eV is observed. This value is much smaller than the one found in Cr­(imz)_2_, which is attributed to the extended conjugation of DAind compared to imz.^60,61^ This highlights the potential of rational chemical design of organic linkers, since it allows engineering MOFs with tunable band gaps from insulators to narrow band gap semiconductors. Interestingly, AM splitting in Cr­(DAind)_2_ is observed along the M−Γ–M′ high-symmetry path (Figure 4e), which is a completely different picture to the one observed in Cr­(imz)_2_. In this case, we observe a d-wave anisotropy of the AM splitting, since only two nodal planes are observed at the AM splitting in the 2D lattice (Figure 4f). We attribute this behavior, which at first sight appears incompatible with the ideal ligand symmetry, to the redistribution of charge and spin density within the DAind linker. In particular, the spin density becomes accumulated in regions close to the magnetic Cr centers with the same spin polarization, as shown in Figure 4c. This redistribution lowers the effective point group symmetry of the ligand from *C*_2v_ and reduces the lattice space group to *Pba*2. As a consequence, the combined symmetries [*E*||*C*_4_], [*C*_2_||*g*_*xy*_], and [*C*_2_|| $g_{x\mathrm{y̅}}$ ] are broken, while the symmetries [*C*_2_||*g*_*x*_] and [*C*_2_||*g*_*y*_] are preserved, as detailed in Section 5 of the Supporting Information. The preservation of these latter symmetry operations maintains the equivalence between the two magnetic sublattices, preventing ferrimagnetism and resulting in a d-wave AM electronic band structure. The magnetic model employed here implicitly assumes that the ligand-symmetry-breaking temperature (*T*_sym_), associated with the onset of ligand spin polarization, lies above the Néel temperature (*T*_N_). According to our HSE06 calculations, in Cr­(DAind)_2_, we observe an AM spin splitting of 83.9 and 35.2 meV in the region close to the Fermi level in the VB and CB, respectively.

### Magnetic Properties of 2D AM MOFs

The distribution of magnetic moments across a lattice gives rise to multiple competing magnetic configurations and a thorough understanding of their relative energies is essential to determine the magnetic ground state of a system. Accordingly, we calculate the energy difference between FM, ferrimagnetic (FiM), and AM configurations in the 2D MOFs studied in this work employing HSE06 (Table 1) and PBE + U functionals (see Supporting Information Sections 1–4). For systems with nonpolarized organic linkers, such as Cr­(imz)_2_ and Cr­(DApent)_2_, a FiM configuration does not exist because the magnetic moments on the Cr atoms are identical. In both materials, the AM configuration constitutes the ground state and is separated from the competing FM state by a substantial energy gap.

**Table 1:** Relative Energies of Different Magnetic Configurations FM, FiM, and AM (in meV/Cr Atom), Magnetic Moment in Cr Atoms and Ligands (*M*_Cr_ and *M*_L_, in μ_B_), Magnetic Exchange Interactions (*J*, in meV), and Magnetic Anisotropy (*D*, in μeV/Cr Atom)

	imz	tdz	DApent	DAind
FM	8.40	290.25	15.14	157.57
FiM	–	0.00	–	2.41
AM	0.00	293.76	0.00	0.00
*M*_Cr_	3.71	3.65	3.65	3.64
*M*_L_	–	0.56	–	0.48
*J*_1_	–1.85	1.72	–3.20	1.45
*J*_2_	–	–22.62	–	3.34
*J*_2′_		–22.62		–13.37
*D*	406.51	377.57	376.12	205.90

A different scenario is observed in 2D MOFs with spin-polarized organic linkers. Here, magnetic moments of 0.56 and 0.48 μ_B_ are observed on the tdz and DAind ligands, respectively, while the total magnetic moment per unit cell remains strictly zero within numerical accuracy. The ligand spin polarization follows the AFM order of the metal sublattices and does not give rise to an independent magnetic sublattice. Calculated magnetic moments of Cr centers remain in a narrow range of 3.6–3.7 μB for all 2D MOFs (Table 1), consistent with a high-spin Cr­(II) electronic configuration. The spin polarization on these radical ligands exhibits a strong tendency to couple antiferromagnetically with metal spins, which has been observed in similar pyz-based MOFs.^27,28^ The dominant role of metal–ligand interactions over metal–metal interactions dictates that magnetic ordering is largely governed by the former. As a result, both the FiM and AM configurations are stabilized to a much greater extent than the FM configuration. Figure 5a shows the magnetic exchange interactions (*J*) between neighboring sites. Here, *J*_1_ denotes the metal–metal magnetic interaction mediated by the organic linkers. When spin polarization is induced on the ligands, additional metal–ligand exchange arises along the lattice (*J*_2_). This interaction is further split into *J*_2_ and *J*_2_′, depending on whether the radical couples ferromagnetically or antiferromagnetically with the neighboring metal, respectively. We employ this notation and the results presented in Table 1 to map a spin Hamiltonian as follows:

1 $$H=-\sum_{i\neq j}J_{ij}\vec{S_{i}}\cdot \vec{S_{j}}-\sum_{i}D_{i}{\left(S_{i}^{z}\right)}^{2}$$

where *J*_*ij*_ represents the magnetic interaction between two magnetic moments along the lattice (*S*_*i*_ and *S*_*j*_) and *D* is the uniaxial magnetic anisotropy, with the easy axis taken perpendicular to the MOF plane. In this expression, a positive (negative) value of *J* favors FM (AFM) coupling between spin moments.

**Figure 5:**
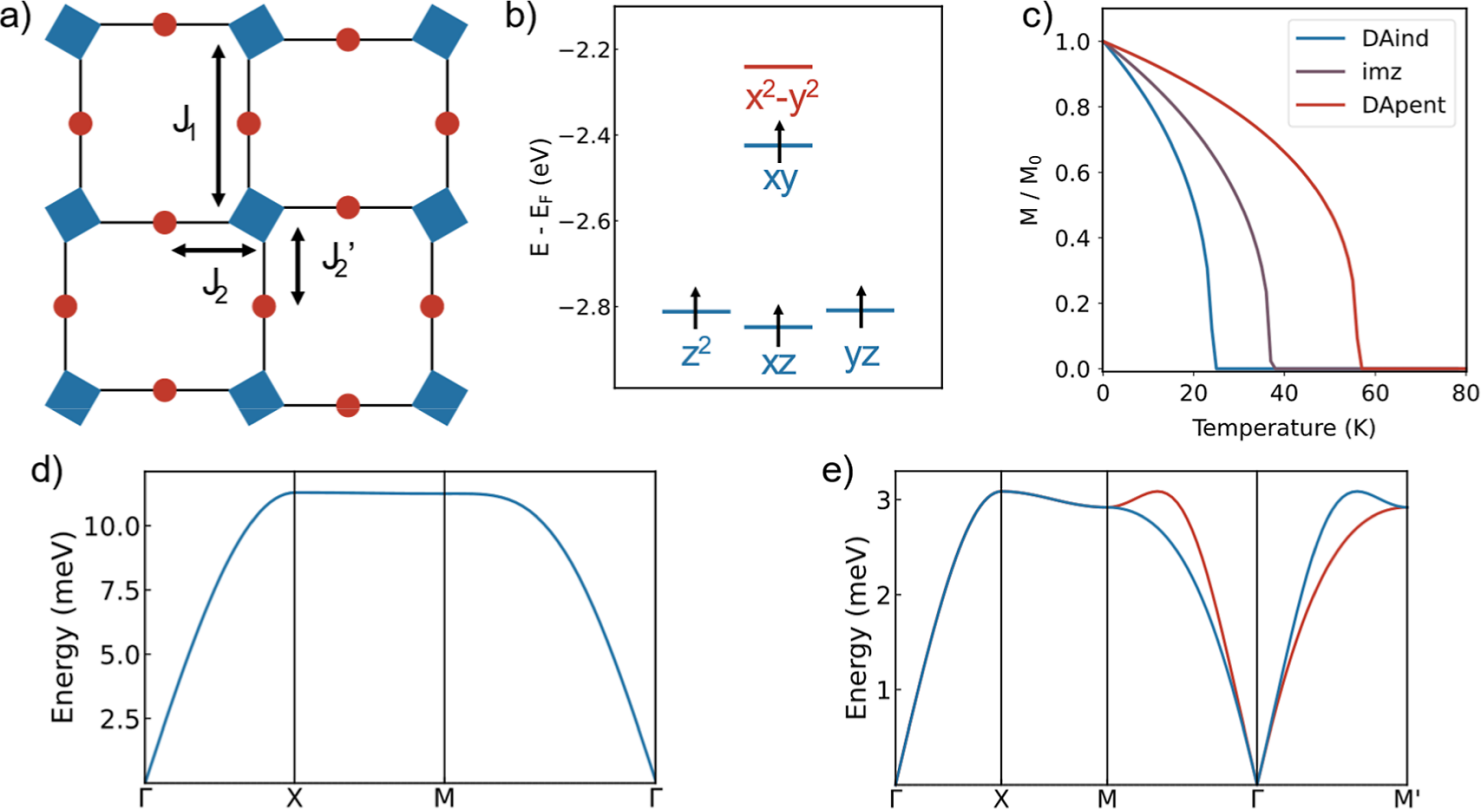
(a) Schematic representation of magnetic interactions in imz-based 2D MOFs. Metal centers are represented as blue squares and ligands as red dots. (b) d orbital energy alignment of Cr atoms in Cr­(DAind)_2_. (c) Calculated *T*_N_ in Cr­(imz)_2_, Cr­(DApent)_2_, and Cr­(DAind)_2_ via atomistic spin dynamics simulations. Simulated spin-wave spectra in (d) g-wave AM Cr­(imz)_2_ and (e) d-wave AM Cr­(DAind)_2_. Color code: blue (red) lines represent left-handed (right-handed) magnons with negative (positive) chirality.

As one can observe in Table 1, in the case of Cr­(tdz)_2_, the FiM configuration is the ground state, which is stabilized due to the dominant AFM contribution of *J*_2_. Thus, no splitting is observed between *J*_2_ and *J*_2_′ since FiM configuration allows symmetric AFM metal–ligand interactions. A net magnetization is present in the Cr­(tdz)_2_ MOF due to FiM coupling between metals and radicals, which is not compatible with altermagnetism. A different scenario is observed in Cr­(DAind)_2_, where AM configuration is the lowest energy magnetic ordering. In this case, the alternated spin polarization over the metal–ligand magnetic structure generates symmetry breaking between *J*_2_ and *J*_2_′ (see Supporting Information Section 7). AFM metal–ligand coupling leads to large negative *J*_2_′, which results in the stabilization of AM order in Cr­(DAind)_2_. Interestingly, *J*_2_ is FM but presents lower intensity than *J*_2_′. We attribute the disparity in the magnitude of *J*_2_ and *J*_2_′ primarily to an asymmetric spin density distribution in DAind (Figure S31) and the specific orbital ordering of the high-spin quasi-square planar Cr­(II) center. Figure 5b illustrates the calculated energy alignment of the d orbitals in Cr­(DAind)_2_, revealing that the $d_{x^{2}-y^{2}}$ orbital remains unoccupied, a configuration that fosters the FM interaction observed in *J*_2_. This interaction through an empty orbital leads to less electronic repulsion and a closer metal–ligand distance. This interpretation is corroborated by bond length analysis, which exhibits a shorter metal–ligand bond length for the FM pathway compared to the slightly larger one (∼0.5%) associated with the AFM interaction. Additionally, we compute *J*_2_ via cluster DFT calculations using a broken symmetry approach to corroborate its remarkable high intensity (see Supporting Information Section 6.5). Broken symmetry calculations align with periodic DFT calculations since we also observe high-intensity magnetic interactions and enhanced *J*_2_ in Cr­(tdz)_2_ compared to Cr­(DAind)_2_.

Next, we calculate *D* as the energy difference between a spin aligned within the MOF plane and aligned out of the plane configurations. In all systems, the preferred orientation is out of plane, with only minor ligand-dependent variations. This trend is consistent with previous *D* calculations on planar tetracoordinated Cr-based 2D MOFs, where an orthogonal spin orientation is also favored.^42,47^ Figure 5c presents the *T*_N_ obtained from atomistic spin-dynamics simulations of the representative 2D AM MOFs described in this work. Notably, the simulated *T*_N_ of Cr­(DApent)_2_ lies close to liquid nitrogen temperature (77 K), reinforcing its potential for AM-based cryogenic spintronic applications. In contrast, the calculated *T*_N_ values for Cr­(DAind)_2_ and Cr­(imz)_2_ are significantly lower, mainly due to the competing FM *J*_1_ in Cr­(DAind)_2_ and the limited strength of magnetic interactions in Cr­(imz)_2_. We note that the calculated *T*_N_ should be interpreted as finite-size crossover temperatures. Explicit simulations performed with and without magnetic anisotropy show only a weak dependence on the anisotropy strength, indicating that exchange interactions dominate the magnetic stabilization in these 2D MOFs (Figures S8, S27, and S37).^62^ Spin wave dispersions in both d-wave and g-wave AM MOFs show linear dispersion the near Γ-point, typical for AFM materials (Figure 5d,e). Interestingly, magnon dispersion in Cr­(DAind)_2_ displays chirality-based spin splitting consistent with d-wave AM anisotropy. On the other hand, in the case of g-wave Cr­(imz)_2_, the spin-Hamiltonian is controlled by the nearest neighbor metal–metal exchange interactions, which increases the effective symmetry and results in degenerate magnon modes, despite the presence of electronic AM spin splitting. This reflects the absence of inequivalent exchange pathways in the spin model (see Supporting Information Section 9).

### Spin-Dependent Transport Simulations

The spin-splitting effect in AM materials is a nonrelativistic analogue of the conventional spin Hall effect in which an applied electric field separates spin-up and spin-down charge currents.^63^ In d-wave AM anisotropy, this effect can be captured by a spin-dependent conductivity tensor. On the other hand, in g-wave AM, symmetry forbids distinct conductivity tensors for different spin component electrons in the linear regime. However, it was recently shown that a spin-splitting effect can nevertheless emerge as a nonlinear response, specifically in the current contribution proportional to the cube of the electric field.^64^ To investigate this behavior, we compute the nonlinear transport response within the Boltzmann formalism, including terms up to the third order in the electric field (see Supporting Information Section 10). Figure 6a shows the off-diagonal component of the conductivity tensor in d-wave Cr­(DAind)_2_ as a function of Fermi energy, evaluated at 300 K and a relaxation time of 10 fs. When the Fermi energy lies in either the conduction or valence band, σ_*yx*_ acquires opposite signs for spin-up and spin-down electrons, resulting in nonrelativistic spin-splitting angles of up to 0.4 degrees. Figure 6b displays the σ_*yxxx*_ component of the nonlinear conductivity tensor in g-wave Cr­(imz)_2_, which gives rise to nonlinear spin splitting.

**Figure 6:**
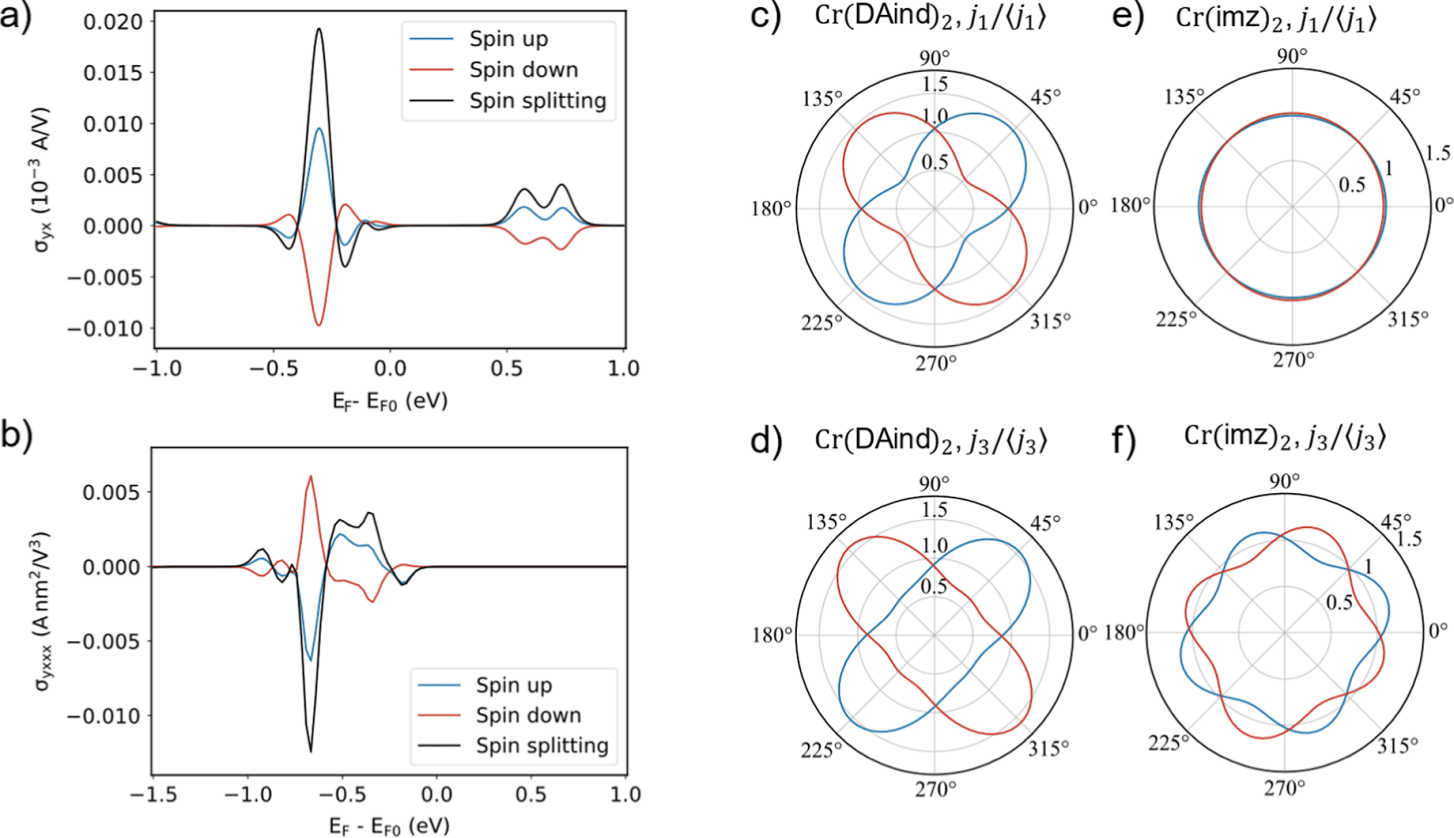
(a) Linear spin-splitting effect in Cr­(DAind)_2_: difference between the off-diagonal components σ_*yx*_ of the conductivity tensor for spin-up and spin-down electrons as a function of the Fermi energy. (b) Third-order spin-splitting effect in Cr­(imz)_2_: difference between the σ_*yxxx*_ components of the nonlinear conductivity tensor for spin-up and spin-down electrons as a function of the Fermi energy. (c–f) Dependence of the current density along the direction of the applied electric field on the field polar angle. Results are shown for Cr­(DAind)_2_ at *E*_F_ – *E*_F_(0) = −0.31 eV (c,d) and for Cr­(imz)_2_ at *E*_F_ – *E*_F_(0) = –0.67 eV (e,f). Panels (c) and (e) show the linear contribution *j*_1_, while panels (d) and (f) show the cubic contribution *j*_3_. Angle brackets denote averaging over the polar angle.

To further analyze spin-dependent transport, Figure 6c–f shows the polar-angle dependence of the spin-up and spin-down currents measured along the direction of the applied field at the Fermi level positions corresponding to the strongest spin splitting (−0.31 eV for Cr­(DAind)_2_ and −0.67 eV for Cr­(imz)_2_). Finally, we separately plot the first-order and third-order contributions in the electric field. In Cr­(DAind)_2_, both contributions follow the expected d-wave symmetry: the conductivity for spin-up electrons is higher along the ΓM direction, whereas for spin-down electrons, it is higher along the ΓM′ direction. For Cr­(imz)_2_, the linear-response transport is spin-independent, but the third-order contribution exhibits the characteristic g-wave symmetry, as shown in Figure 6f. In this context, the spin-dependent transport calculations presented here should be viewed as symmetry-based predictions of the transport responses expected once sufficient electronic delocalization is achieved. We note that the MOFs investigated in this work are semiconducting in their neutral state and that experimental access to the predicted transport effects would require shifting the Fermi level away from the middle of the band gap. Such control of the Fermi level has been demonstrated in a broad range of MOFs through chemical doping, redox-active ligands, electrostatic gating, or interface engineering, as discussed extensively for electrically conductive MOFs.^65,66^ These results therefore provide direct guidance toward future metallic or near-metallic realizations of AM MOFs, where the predicted spin-dependent transport effects could be accessed experimentally.

## Conclusions

In summary, we present a general design strategy for engineering AM nonrelativistic spin splitting in 2D MOFs through ligand-symmetry-driven lattice symmetry breaking. We show that modifying the symmetry of the coordination network through the choice and arrangement of the organic linkers introduces an inequivalence between the square-planar Cr sublattices, generating a sizable g-wave AM spin splitting of 65.1 meV in Cr­(imz)_2_. This demonstrates that coordination chemistry alone can be systematically used to tailor altermagnetism in low-dimensional molecular materials. On leveraging the broad chemical space of organic ligands, we further construct related 2D MOFs where increased π-conjugation enables systematic control over both electronic structure and magnetic behavior. We then apply FMOE to selectively induce spin polarization on the ligand scaffold in imz-derived MOFs. The emergence of ligand-localized magnetic moments breaks the sublattice equivalence and drives a transition to d-wave AM anisotropy, reaching spin splitting up to 83.9 meV in Cr­(DAind)_2_. In these systems, strong metal–ligand interactions stabilize the magnetic ground state and preserve the AM phase. Interestingly, we show that AM spin splitting manifests not only in the electronic band structure but also in the spin-wave spectrum, where chiral magnon splitting is observed in d-wave Cr­(DAind)_2_. Finally, we show that AM spin splitting directly manifests in charge transport as a nonrelativistic analogue of the spin Hall effect. In d-wave Cr­(DAind)_2_, spin-dependent linear conductivity leads to sizable spin-splitting angles, while in g-wave Cr­(imz)_2_, symmetry forbids linear spin separation but allows a distinct nonlinear response emerging at the third order in the electric field. Importantly, both regimes generate measurable spin-polarized currents under experimentally realistic conditions, making nonlinear transport a viable probe of altermagnetism in 2D MOF monolayers. Together, these results demonstrate that coordination chemistry provides a powerful and versatile route to design 2D MOFs with tunable AM properties, opening new opportunities for chemically engineered spin functionalities in coordination solids.

## Methods

Structural relaxations, electronic structure, magnetic configurations, AIMD and phonon calculations are performed using the VASP package.^67^ We employ the generalized gradient approximation (GGA) to describe the exchange–correlation energy, in combination with the PBE functional. Given the close interaction between adjacent ligands, we employ Grimme D3 vdW corrections. We introduce a vacuum layer of 15 Å to avoid periodic interactions between 2D layers. To accurately describe the partially filled 3d orbitals of Cr in GGA-PBE calculations, we use Hubbard U correction (see Supporting Information for DFT + U results). To accurately describe the electronic and magnetic properties, we calculate the electronic band structures and the relative energy of each magnetic configuration using the hybrid HSE06 functional (see Supporting Information for comparison with DFT + U results). The projector augmented-wave (PAW) pseudopotential in combination with a plane-wave cutoff of 500 eV is used. A Monkhorst–Pack *k*-point mesh of 3 × 3 × 1 (2 × 2 × 1) is used for monocyclic (polycyclic)-based 2D MOFs in HSE06 calculations and a *k*-point mesh of 5 × 5 × 1 (3 × 3 × 1) in PBE + U calculations. We perform spin-polarized geometrical optimization with a convergence criterion of 0.01 eV/Å. The symmetry of the relaxed crystal structures was analyzed using a Python code, which determines the crystallographic space group within a given numerical tolerance and evaluates its compatibility with AM symmetry conditions.^68^ AIMD simulations are performed for 5 ps with a time step of 1 fs at 300 and 600 K using the canonical *NVT* ensemble. Phonon dispersions are calculated using Phonopy code.^69^ Atomic charge analysis is performed using Bader algorithm.^70^ Magnetic parameters demanded to map the spin Hamiltonian (eq 1) are calculated using SIESTA software,^71,72^ in order to take benefit from its localized atomic orbital approach. We use PBE + U (*U*_eff_ = 3 eV) in combination with the double-ζ polarized basis set and a real-space mesh cutoff of 900 Ry. SOC is considered for *D* calculations, where we analyze the energy difference between in-plane and out-of-plane spin orientations. Magnetic interactions (*J*) are calculated using the interface between SIESTA and TB2J code.^73^ Cluster DFT calculations are performed using Gaussian09 software in its revision D01,^74^ where we employ the B3LYP functional in combination with the Def2TZVP basis set. Maximally localized Wannier functions are constructed using Wannier90,^75^ with the d orbitals of Cr, the s and p orbitals of C, the p orbitals of N, and the s orbitals of H as the basis, to generate a tight-binding Hamiltonian for spin-dependent transport calculations. Transport calculations are made within Boltzmann formalism with a single phenomenological relaxation time (τ) of 10 fs and a temperature of 300 K.

## Supplementary Material



## Notes

Additional calculations; symmetry analysis; cluster DFT calculations; SIESTA calculations; Bader charge analysis; linear spin-wave theory simulations; and spin-dependent transport calculations (PDF)
