# Supplementary material for: Chemical Engineering of Altermagnetism in Two-Dimensional Metal–Organic Frameworks
Source: J Am Chem Soc. 2026 May 14;148(25):25415–25. doi: 10.1021/jacs.5c22589 (PMC13339145; doi:10.1021/jacs.5c22589)
Supplement: Supplementary file 1 [file ja5c22589_si_001.pdf]

# Supporting Information for Chemical Engineering of Altermagnetism in Two-Dimensional Metal-Organic Frameworks

*Diego López-Alcalá, Alberto M. Ruiz, Andrei Shumilin and José J. Baldoví*.*

Instituto de Ciencia Molecular, Universitat de València, Catedrático José Beltrán 2, 46980 Paterna, Spain.

E-mail: j.jaime.baldovi@uv.es

**Table of Contents**

1. **$Cr(imz)_2$……………………………………………………………2**
2. **$Cr(tdz)_2$……………………………………………………………7**
3. **$Cr(DApent)_2$……..…………………………………………………13**
4. **$Cr(Daind)_2$……..…………………..…………………………….21**
5. **Symmetry analysis of $M(pyz)_2$, $Cr(imz)_2$ and $Cr(DAind)_2$…………………29**
6. **Cluster DFT Calculations……..……………………………………..32**
   - **6.1 imz……………………………………………………………….32**
   - **6.2 tdz……………………………………………………………..…34**
   - **6.3 DApent……………………………………………………….…36**
   - **6.4 DAind………………………………………………………….....39**
   - **6.5 Broken Symmetry Calculations……………………………………..41**
7. **SIESTA Calculations………………………………………………42**
8. **Bader Charge Analysis……………………………………………..45**
9. **Linear Spin-Wave Theory Simulations……………………………..……45**
10. **Spin-Dependent Transport Calculations………..………..………….……...45**

# 1. Cr(imz)$_2$

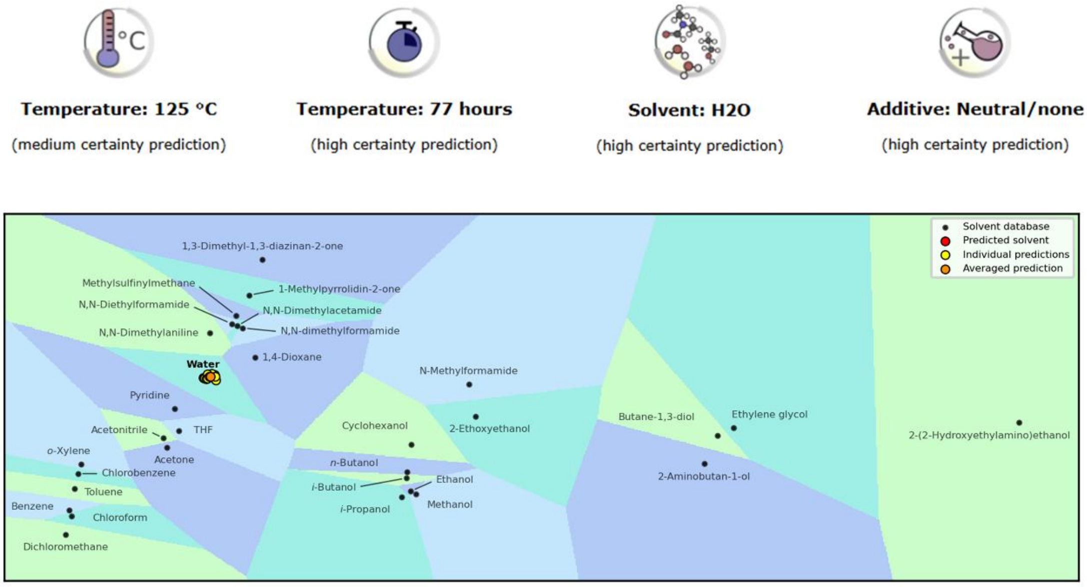


**Figure S1.** Synthesis protocol prediction of Cr(imz)$_2$. Obtained using machine learning tool available at https://mof-synthesis.aimat.science.

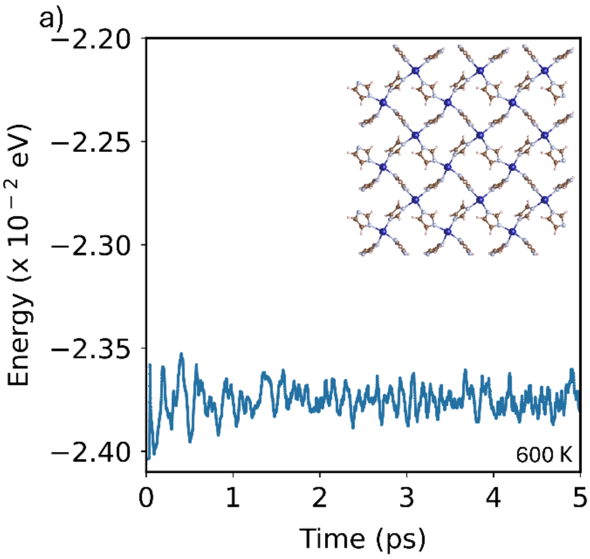


**Figure S2.** AIMD simulation at 600 K in Cr(imz)$_2$. Inset image represents a snapshot of the structure during the simulation.

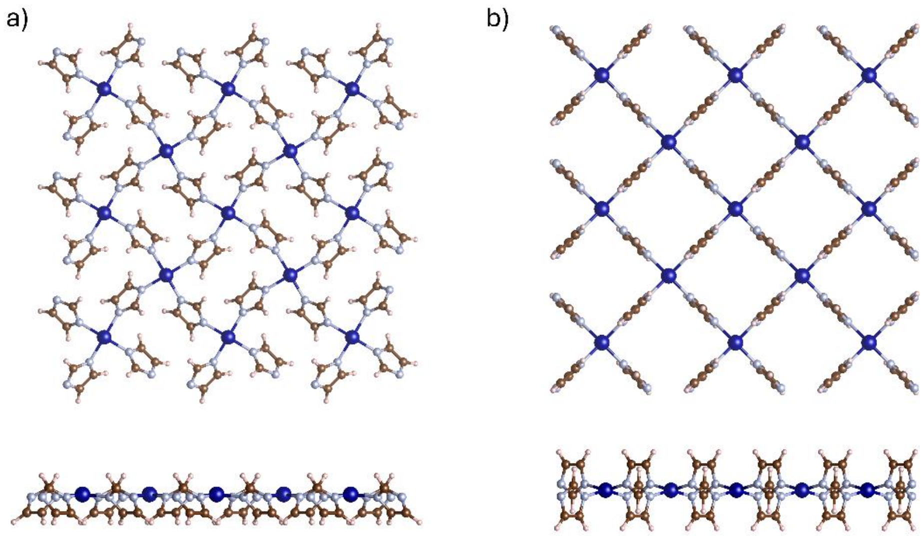


**Figure S3.** Top and side views of a) clockwise and b) alternating ligand-orientation patterns in $Cr(imz)_2$.

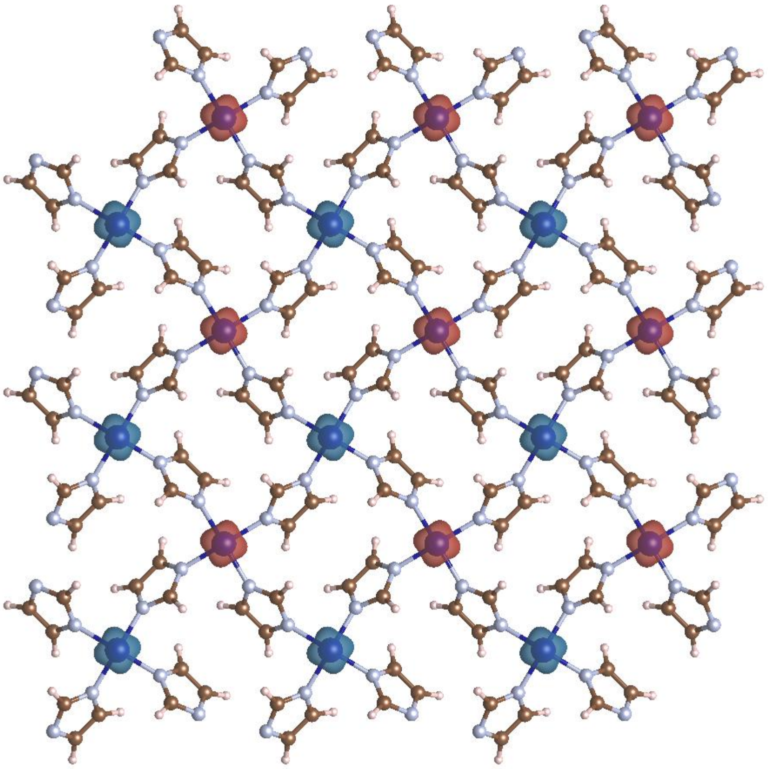

**Figure S4.** Spin density in $Cr(imz)_2$. Blue (red) isosurface represents spin up (down) density.

**Table S1.** Electronic band gap ($E_g$), magnetic moment in Cr atoms and ligand ($M_{Cr}$ and $M_L$ in $\mu_B$) and relative energies of different magnetic configurations FM, FiM and AM (in meV/Cr atom) in $Cr(imz)_2$.

| | **$U_{eff}$ (eV)** | | | |
|---|---|---|---|---|
| | **2** | **3** | **4** | **5** |
| ***$E_g$*** | 3.59 | 3.96 | 4.12 | 4.15 |
| ***$M_{Cr}$*** | 3.66 | 3.71 | 3.75 | 3.80 |
| ***$M_L$*** | - | - | - | - |
| **FM** | 9.43 | 7.52 | 6.08 | 4.96 |
| **FiM** | - | - | - | - |
| **AM** | 0 | 0 | 0 | 0 |

**Table S2.** Comparison of total energy (E, in eV) and AM spin splitting ($E_s$, in meV) in $Cr(imz)_2$ calculated using PBE+U with different k-point mesh.

| **PBE+U** | **k-point mesh** | | |
|---|---|---|---|
| | **3x3x1** | **5x5x1** | **7x7x1** |
| **E** | -238.090743 | -238.090977 | -238.091017 |
| **$E_s$** | 69.8 | 69.8 | 69.8 |

**Table S3.** Comparison of total energy (E, in eV) and AM spin splitting ($E_s$, in meV) in $Cr(imz)_2$ calculated using HSE06 with different k-point mesh.

| **HSE06** | **k-point mesh** | | |
|---|---|---|---|
| | **3x3x1** | **5x5x1** | **7x7x1** |
| **E** | -287.501992 | -287.502199 | -287.502705 |
| **$E_s$** | 65.0 | 65.1 | 65.1 |

**Table S4.** HSE06 calculated atomic magnetic moments in $Cr(imz)_2$ unit cell.

| **Atom** | **Magnetic moment (μB)** |
|---|---|
| Cr1 | 3.706 |
| Cr2 | -3.706 |
| N1 | -0.044 |
| N2 | -0.044 |
| N3 | -0.044 |
| N4 | -0.044 |
| N5 | 0.044 |
| N6 | 0.044 |
| N7 | 0.044 |
| N8 | 0.044 |
| C1 | -0.008 |

| | |
|---|---|
| C2 | -0.008 |
| C3 | -0.008 |
| C4 | -0.008 |
| C5 | 0.008 |
| C6 | 0.008 |
| C7 | 0.008 |
| C8 | 0.008 |
| C9 | 0.000 |
| C10 | 0.000 |
| C11 | 0.000 |
| C12 | 0.000 |
| H1 | 0.000 |
| H2 | 0.000 |
| H3 | 0.000 |
| H4 | 0.000 |
| H5 | 0.000 |
| H6 | 0.000 |
| H7 | 0.000 |
| H8 | 0.000 |
| H9 | 0.000 |
| H10 | 0.000 |
| H11 | 0.000 |
| H12 | 0.000 |
| **Total** | **0.000** |

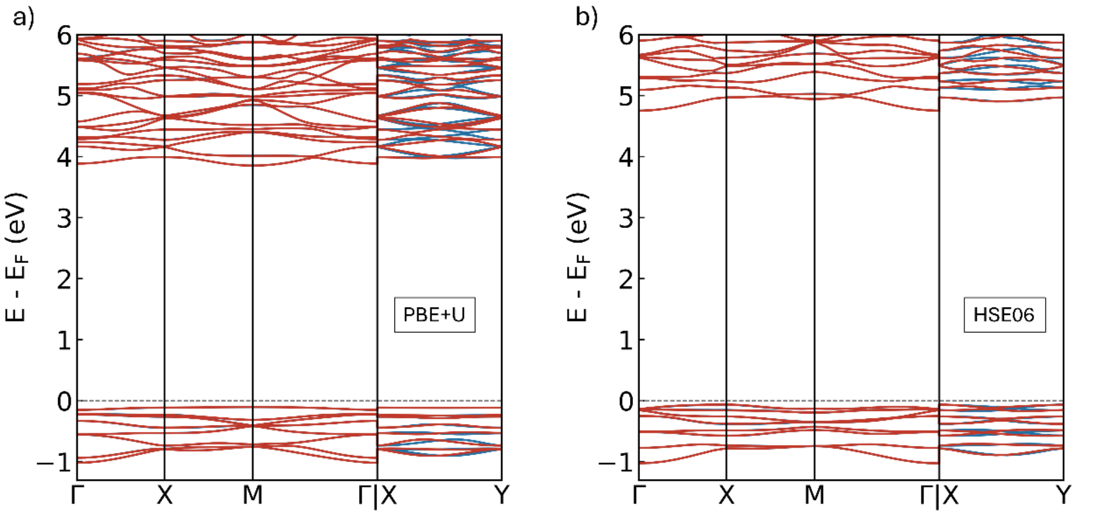


**Figure S5.** Calculated electronic band structure using a) PBE+U ($U_{eff}$ = 3 eV) and b) HSE06 functionals in $Cr(imz)_2$.

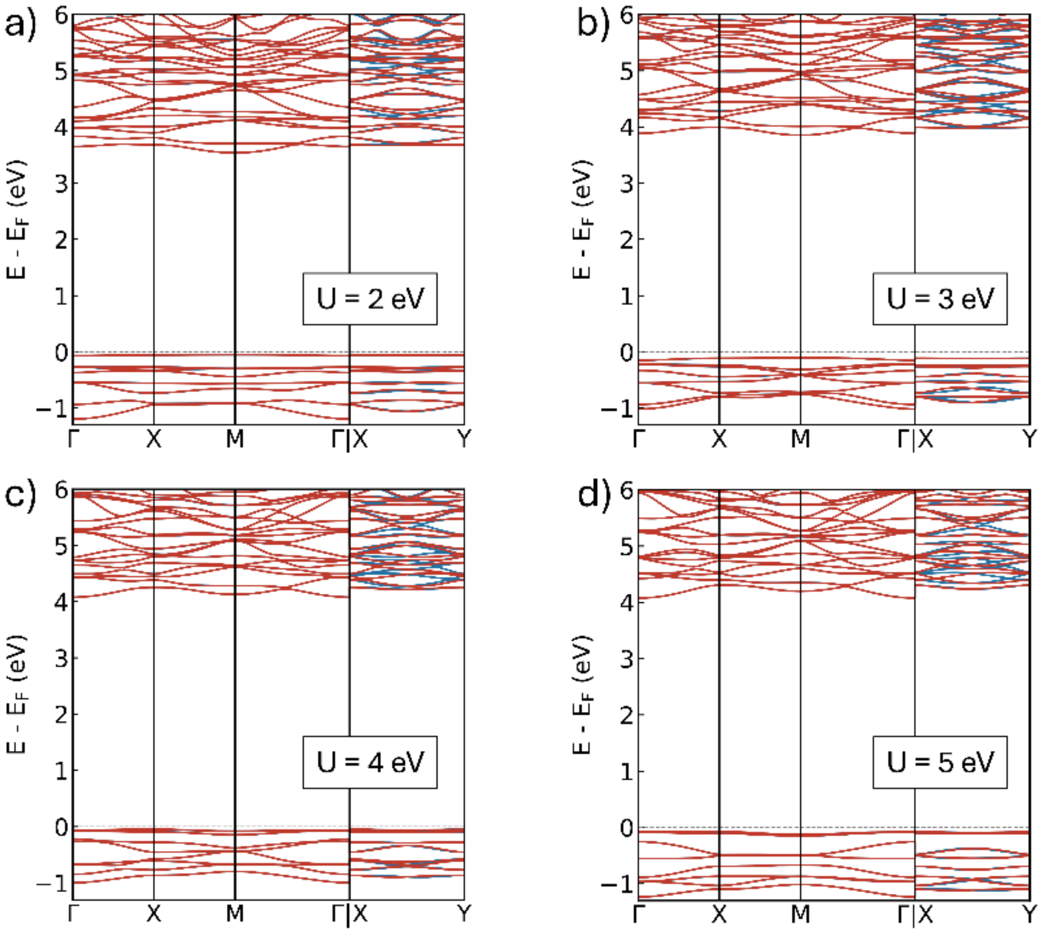


**Figure S6.** Electronic band structure of Cr(imz)$_2$ using PBE+U. a) $U_{eff}$ = 2 eV, b) $U_{eff}$ = 3 eV, c) $U_{eff}$ = 4 eV and d) Ueff = 5 eV.

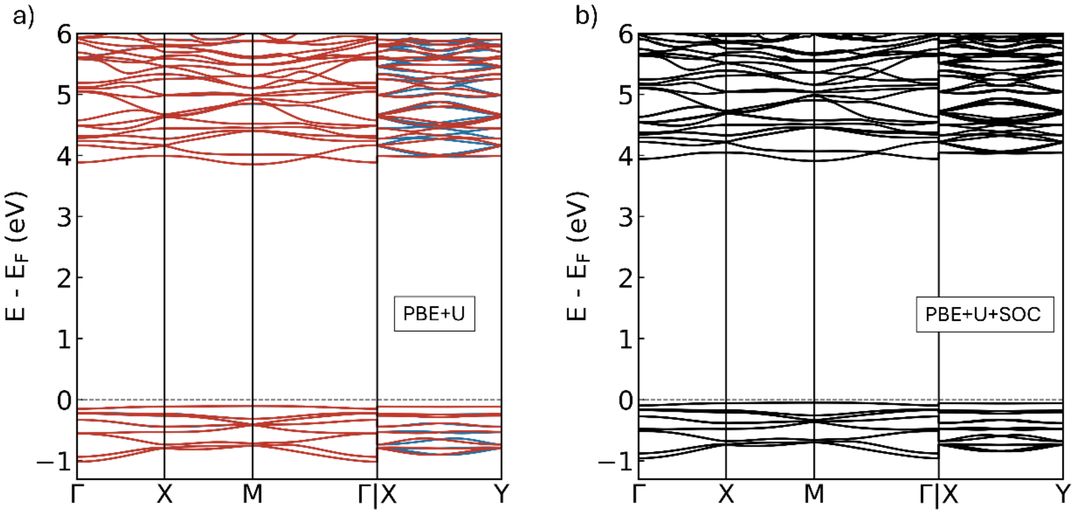


**Figure S7.** Calculated electronic band structure using PBE+U ($U_{eff}$ = 3 eV) a) without SOC and b) with SOC in Cr(imz)$_2$.

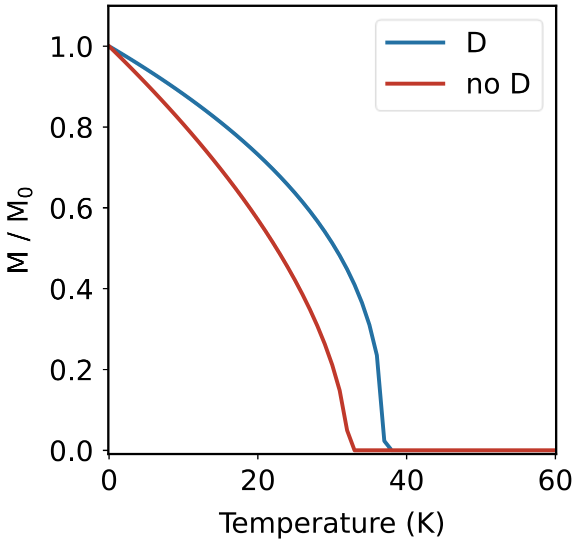


**Figure S8.** Atomistic spin dynamics simulations with (without) considering magnetic anisotropy in $Cr(imz)_2$ in blue (red).

## 2. $Cr(tdz)_2$

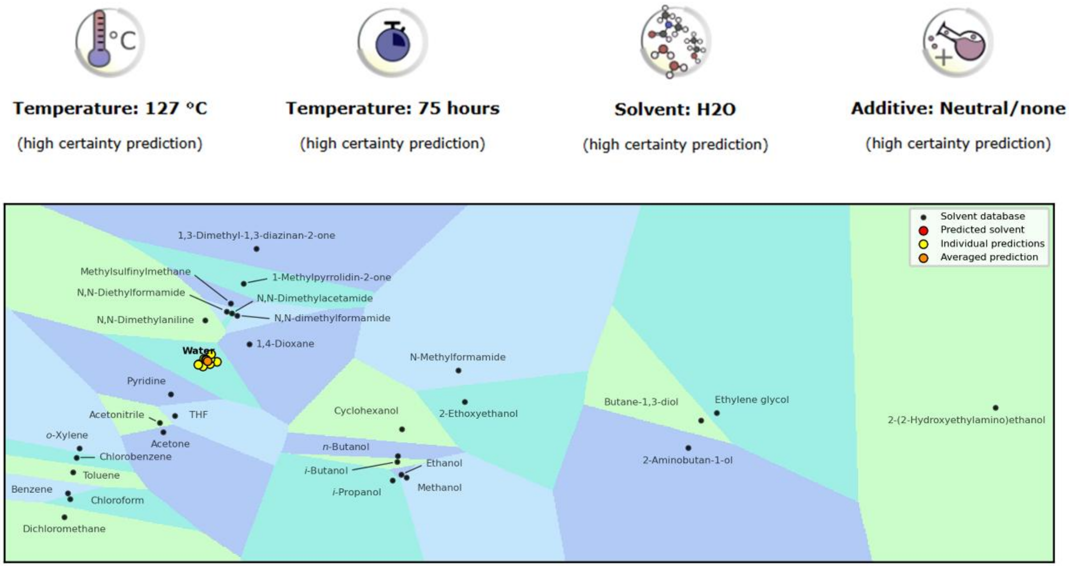


**Figure S9.** Synthesis protocol prediction of $Cr(tdz)_2$. Obtained using machine learning tool available at https://mof-synthesis.aimat.science.

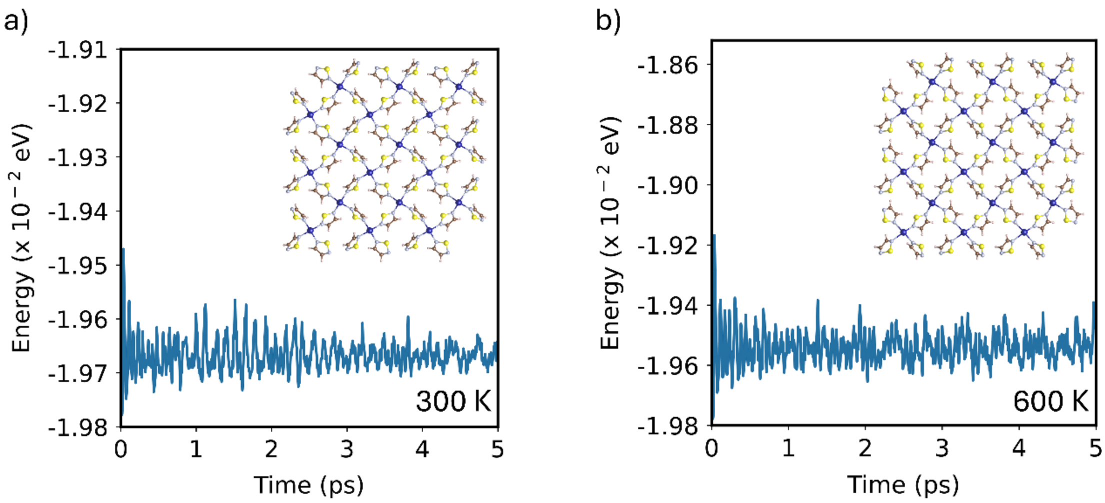


**Figure S10.** AIMD simulations at a) 300 K and b) 600 K in $Cr(tdz)_2$. Inset images represent a snapshot of the structure during the simulation.

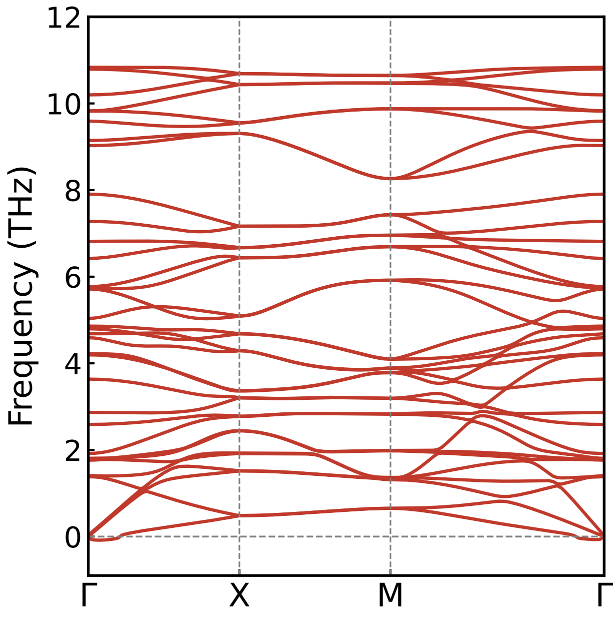


**Figure S11.** Phonon dispersion of $Cr(tdz)_2$.

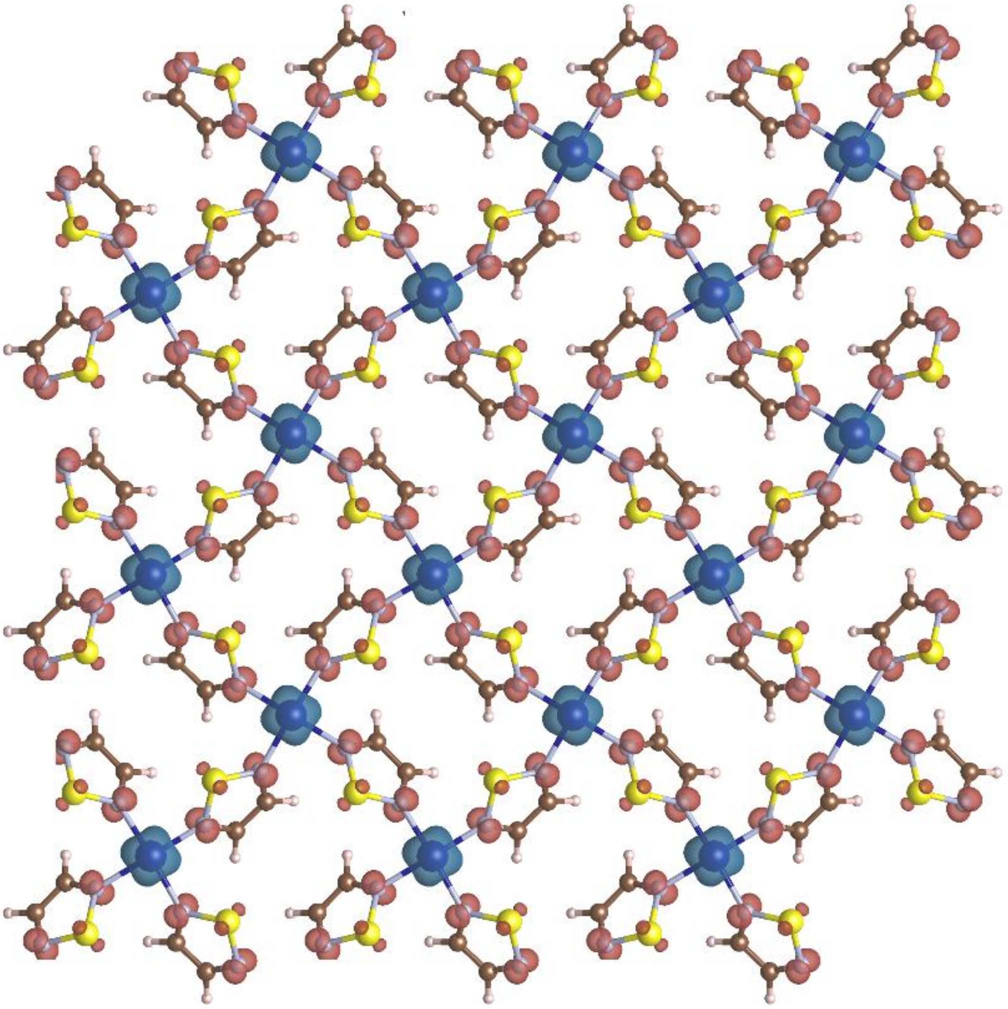

**Figure S12.** Spin density of FiM ordering in $Cr(tdz)_2$.

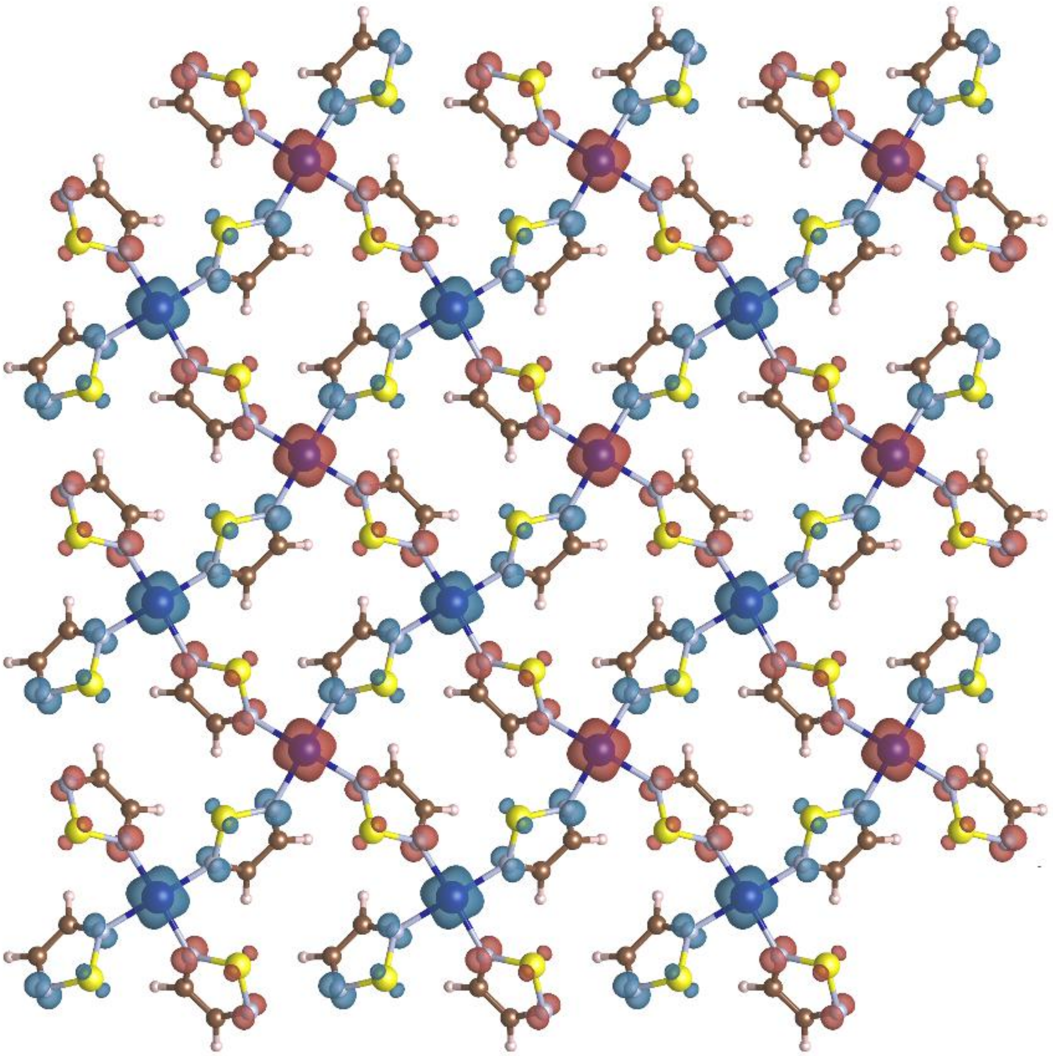

**Figure S13.** Spin density of AM ordering in $Cr(tdz)_2$.

**Table S5.** Electronic band gap ($E_g$), magnetic moment in Cr atoms and ligand ($M_{Cr}$ and $M_L$ in $\mu_B$) and relative energies of different magnetic configurations FM, FiM and AM (in meV/Cr atom) in $Cr(tdz)_2$.

| | **$U_{eff}$ (eV)** | | | |
|---|---|---|---|---|
| | **2** | **3** | **4** | **5** |
| **$E_g$** | 0.50 | 0.51 | 0.51 | 0.51 |
| **$M_{Cr}$** | 3.47 | 3.57 | 3.64 | 3.71 |
| **$M_L$** | 0.55 | 0.57 | 0.58 | 0.60 |
| **FM** | 288.70 | 342.83 | 218.49 | 359.19 |
| **FiM** | 0.00 | 0.00 | 0.00 | 0.00 |
| **AM** | 279.08 | 251.93 | 227.03 | 204.36 |

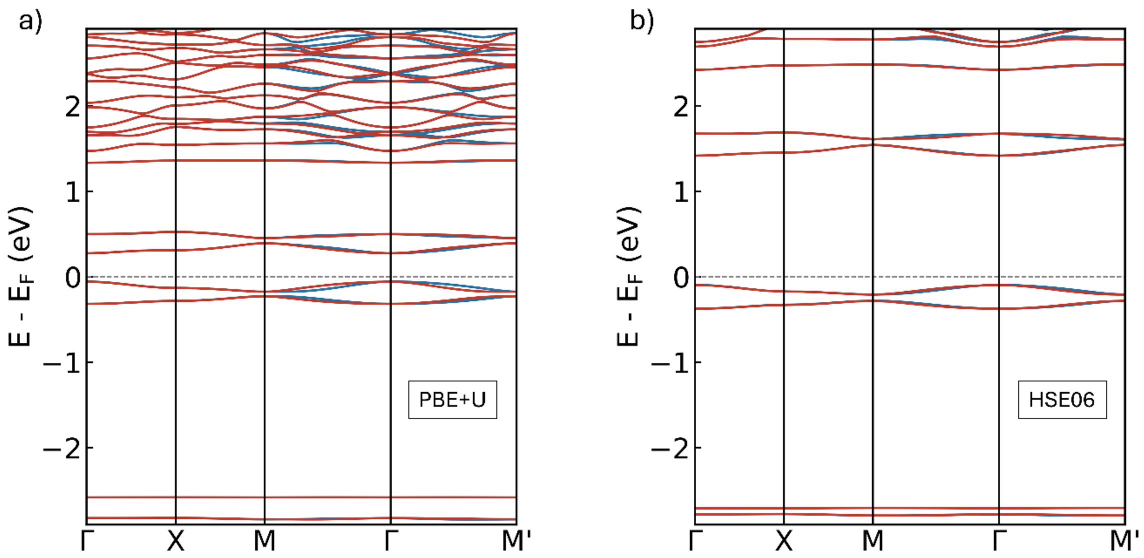


**Figure S14.** Calculated electronic band structure using a) PBE+U ($U_{eff}$ = 3 eV) and b) HSE06 functionals in AM state of $Cr(tdz)_2$.

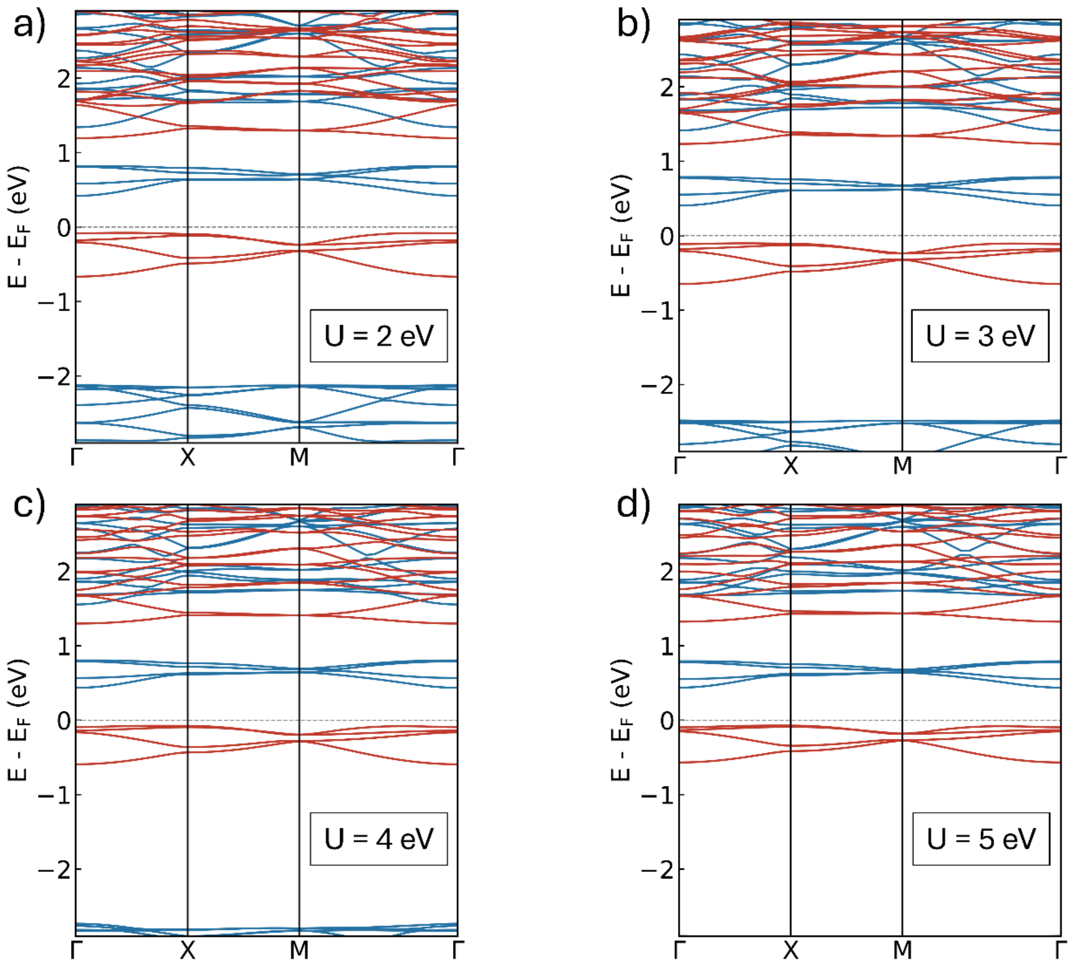


**Figure S15.** Electronic band structure of FiM state in Cr(tdz)₂ using PBE+U. a) $U_{eff}$ = 2 eV, b) $U_{eff}$ = 3 eV, c) $U_{eff}$ = 4 eV and d) Ueff = 5 eV.

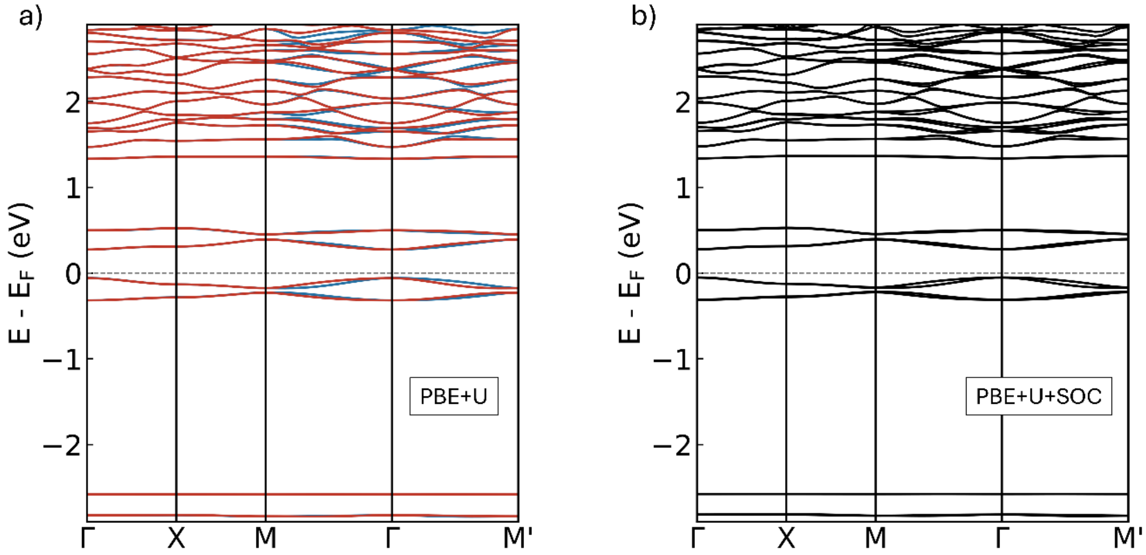


**Figure S16.** Calculated electronic band structure using PBE+U ($U_{eff}$ = 3 eV) a) without SOC and b) with SOC in AM state of $Cr(tdz)_2$.

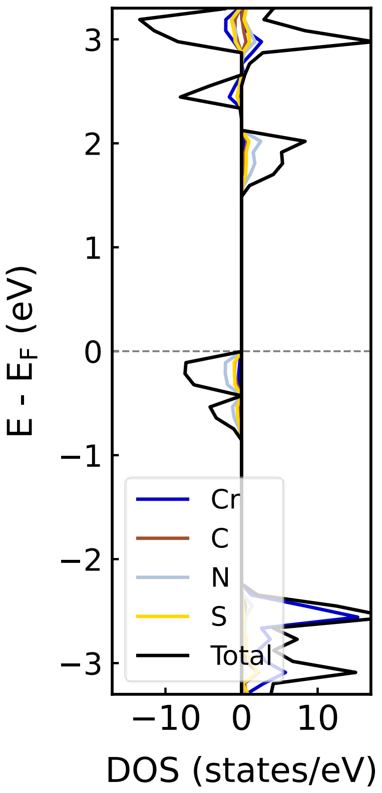


**Figure S17.** PDOS in FiM state of Cr(tdz)$_2$ calculated using HSE06 functional.

## 3. Cr(DApent)$_2$

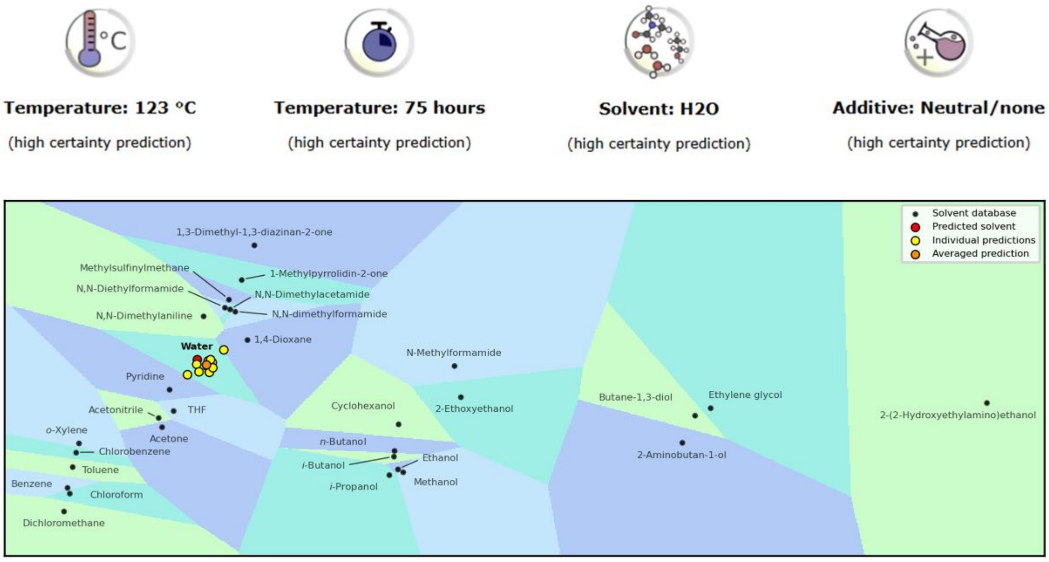


**Figure S18.** Synthesis protocol prediction of Cr(DApent)$_2$. Obtained using machine learning tool available at https://mof-synthesis.aimat.science.

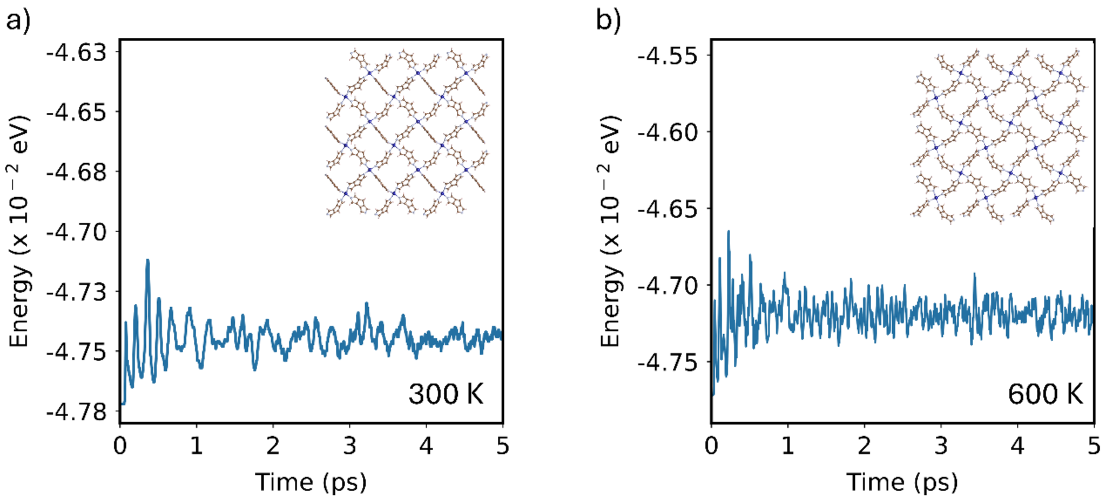


**Figure S19.** AIMD simulations at a) 300 K and b) 600 K in Cr(DApent)$_2$. Inset images represent a snapshot of the structure during the simulation.

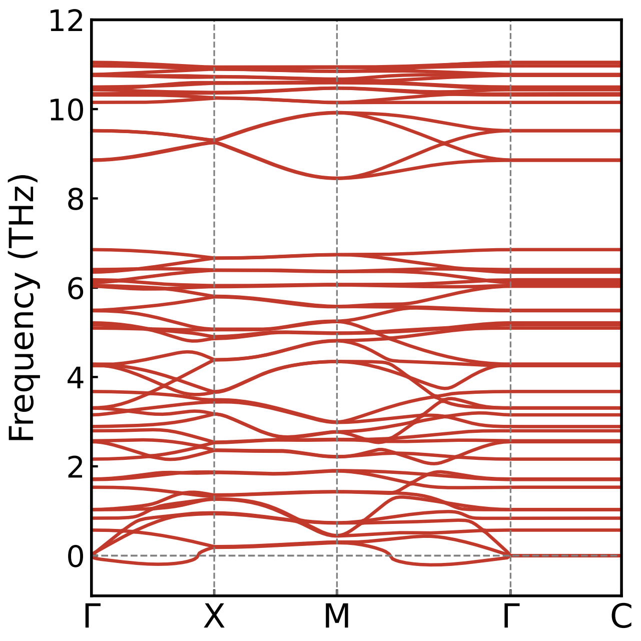


**Figure S20.** Phonon dispersion of Cr(DApent)$_2$.

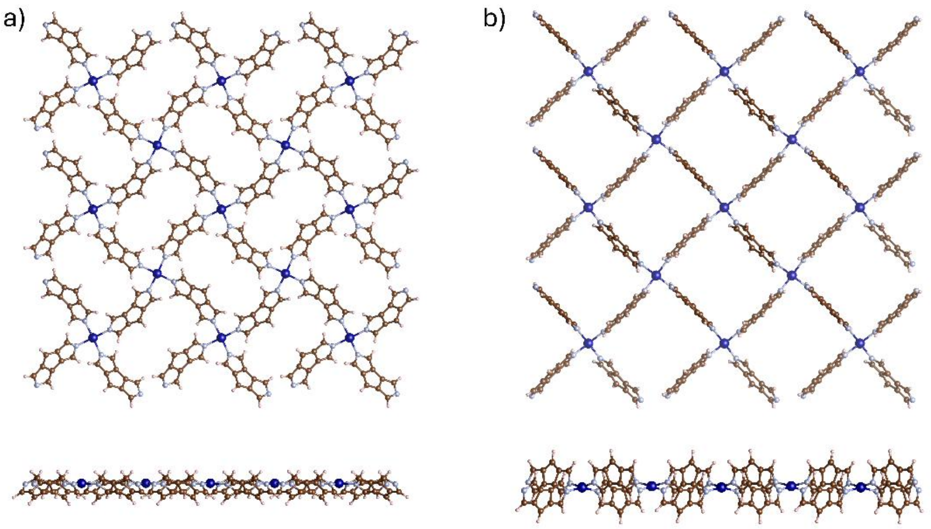


**Figure S21.** Top and side views of a) clockwise and b) alternating ligand-orientation patterns in Cr(DApent)$_2$.

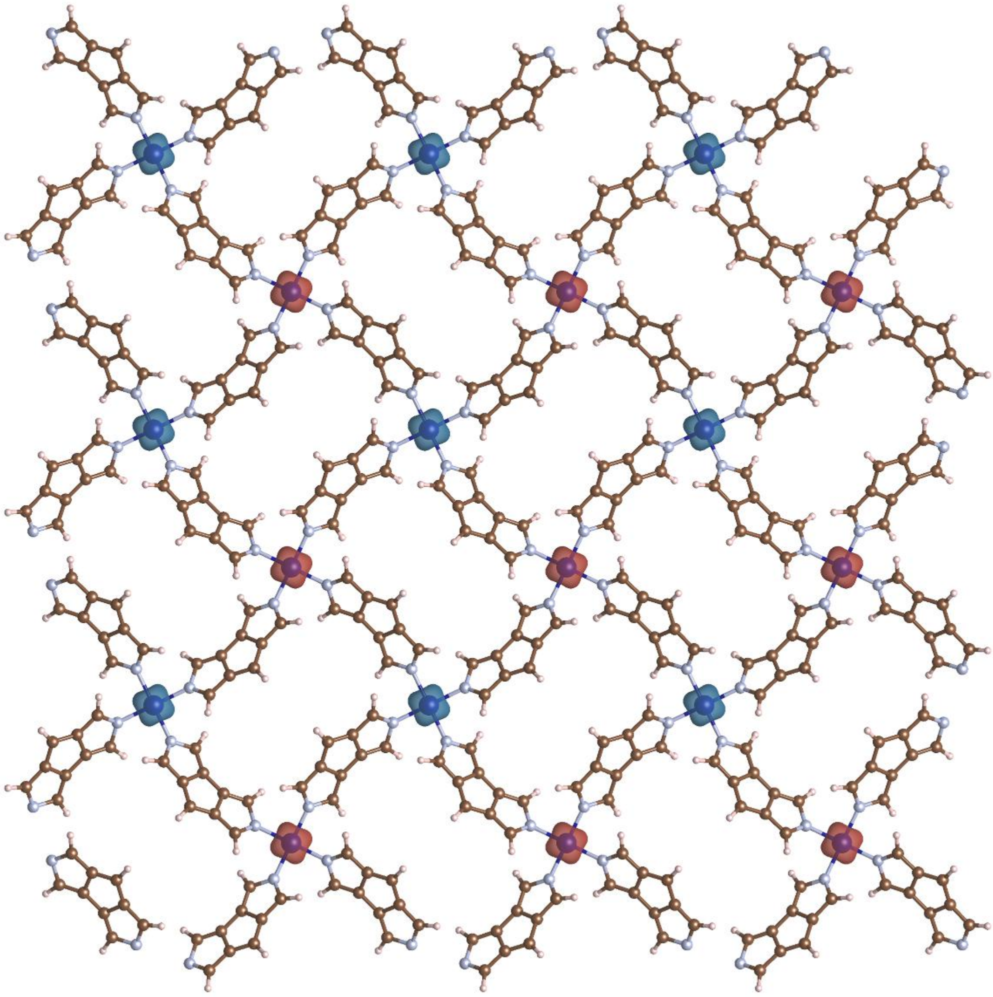

**Figure S22.** Spin density in Cr(DApent)$_2$.

**Table S6.** Electronic band gap ($E_g$), magnetic moment in Cr atoms and ligand ($M_{Cr}$ and $M_L$ in $\mu_B$) and relative energies of different magnetic configurations FM, FiM and AM (in meV/Cr atom) in $Cr(DApent)_2$.

| | **$U_{eff}$ (eV)** | | | |
|---|---|---|---|---|
| | **2** | **3** | **4** | **5** |
| ***$E_g$*** | 0.94 | 0.97 | 0.98 | 0.98 |
| ***$M_{Cr}$*** | 3.58 | 3.65 | 3.71 | 3.77 |
| ***$M_L$*** | - | - | - | - |
| **FM** | 20.11 | 15.15 | 11.53 | 8.86 |
| **FiM** | - | - | - | - |
| **AM** | 0 | 0 | 0 | 0 |

**Table S7.** Comparison of total energy (E, in eV) and AM spin splitting ($E_s$, in meV) in $Cr(DApent)_2$ calculated using PBE+U with different k-point mesh.

| **PBE+U** | **k-point mesh** | | | |
|---|---|---|---|---|
| | **1x1x1** | **2x2x1** | **3x3x1** | **4x4x1** |
| **E** | -477.122599 | -477.12242 | -477.122421 | -477.12242 |
| **$E_s$** | 12.7 | 13.1 | 13.0 | 13.1 |

**Table S8.** Comparison of total energy (E, in eV) and AM spin splitting ($E_s$, in meV) in $Cr(DApent)_2$ calculated using HSE06 with different k-point mesh.

| | **k-point mesh** | | | |
|---|---|---|---|---|
| **HSE06** | **1x1x1** | **2x2x1** | **3x3x1** | **4x4x1** |
| **E** | -562.35593 | -562.396248 | -562.3963 | -562.3963 |
| **$E_s$** | 29.1 | 29.3 | 29.3 | 29.3 |

**Table S9.** HSE06 calculated atomic magnetic moments in $Cr(DApent)_2$ unit cell.

| **Atom** | **Magnetic moment (μB)** |
|---|---|
| Cr1 | 3.657 |
| Cr2 | -3.657 |
| C1 | 0.023 |
| C2 | -0.023 |
| C3 | 0.023 |
| C4 | -0.023 |
| C5 | 0.034 |
| C6 | -0.013 |
| C7 | -0.008 |

| | |
|---|---|
| C8 | 0.013 |
| C9 | 0.008 |
| C10 | 0.000 |
| C11 | -0.034 |
| C12 | 0.034 |
| C13 | -0.013 |
| C14 | -0.008 |
| C15 | 0.013 |
| C16 | 0.008 |
| C17 | 0.000 |
| C18 | -0.034 |
| C19 | -0.023 |
| C20 | 0.023 |
| C21 | -0.034 |
| C22 | 0.013 |
| C23 | 0.008 |
| C24 | -0.013 |
| C25 | -0.008 |
| C26 | 0.000 |
| C27 | 0.034 |
| C28 | -0.023 |
| C29 | 0.023 |
| C30 | -0.034 |
| C31 | 0.013 |
| C32 | 0.008 |
| C33 | -0.013 |
| C34 | -0.008 |
| C35 | 0.000 |
| C36 | 0.034 |
| H1 | 0.000 |
| H2 | 0.000 |
| H3 | 0.000 |
| H4 | 0.000 |
| H5 | -0.001 |
| H6 | 0.000 |
| H7 | 0.001 |
| H8 | -0.001 |
| H9 | 0.000 |
| H10 | 0.001 |
| H11 | 0.000 |
| H12 | 0.000 |
| H13 | 0.001 |
| H14 | 0.000 |
| H15 | -0.001 |
| H16 | 0.000 |

| | |
|---|---|
| H17 | 0.000 |
| H18 | 0.001 |
| H19 | 0.000 |
| H20 | -0.001 |
| N1 | -0.071 |
| N2 | 0.071 |
| N3 | -0.071 |
| N4 | 0.071 |
| N5 | 0.071 |
| N6 | -0.071 |
| N7 | 0.071 |
| N8 | -0.071 |
| **Total** | **0.000** |

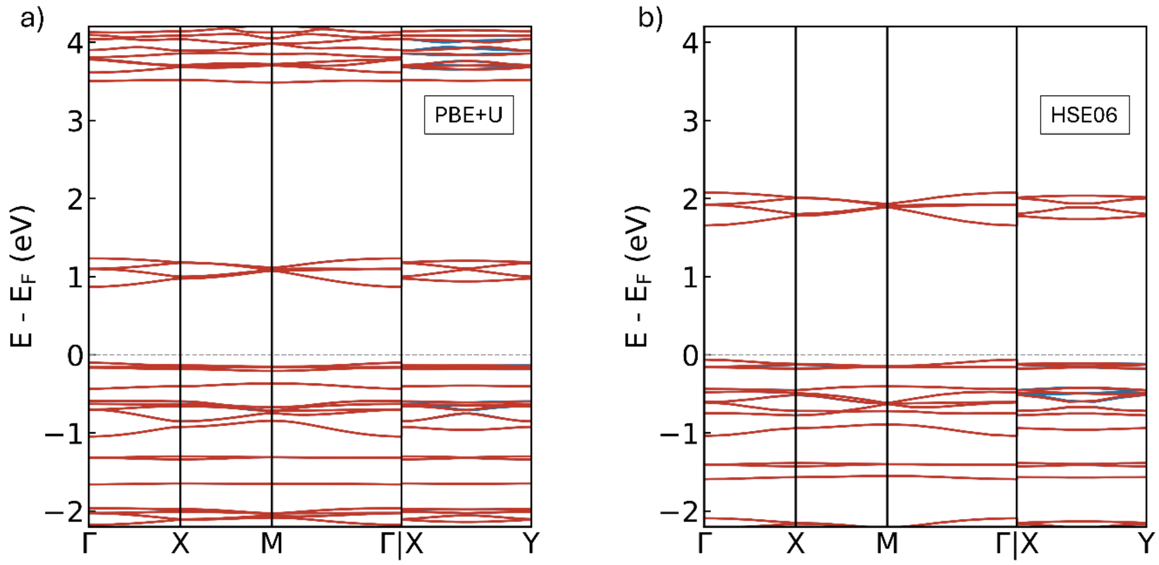


**Figure S23.** Calculated electronic band structure using a) PBE+U ($U_{eff}$ = 3 eV) and b) HSE06 functionals in AM state of $Cr(DApent)_2$.

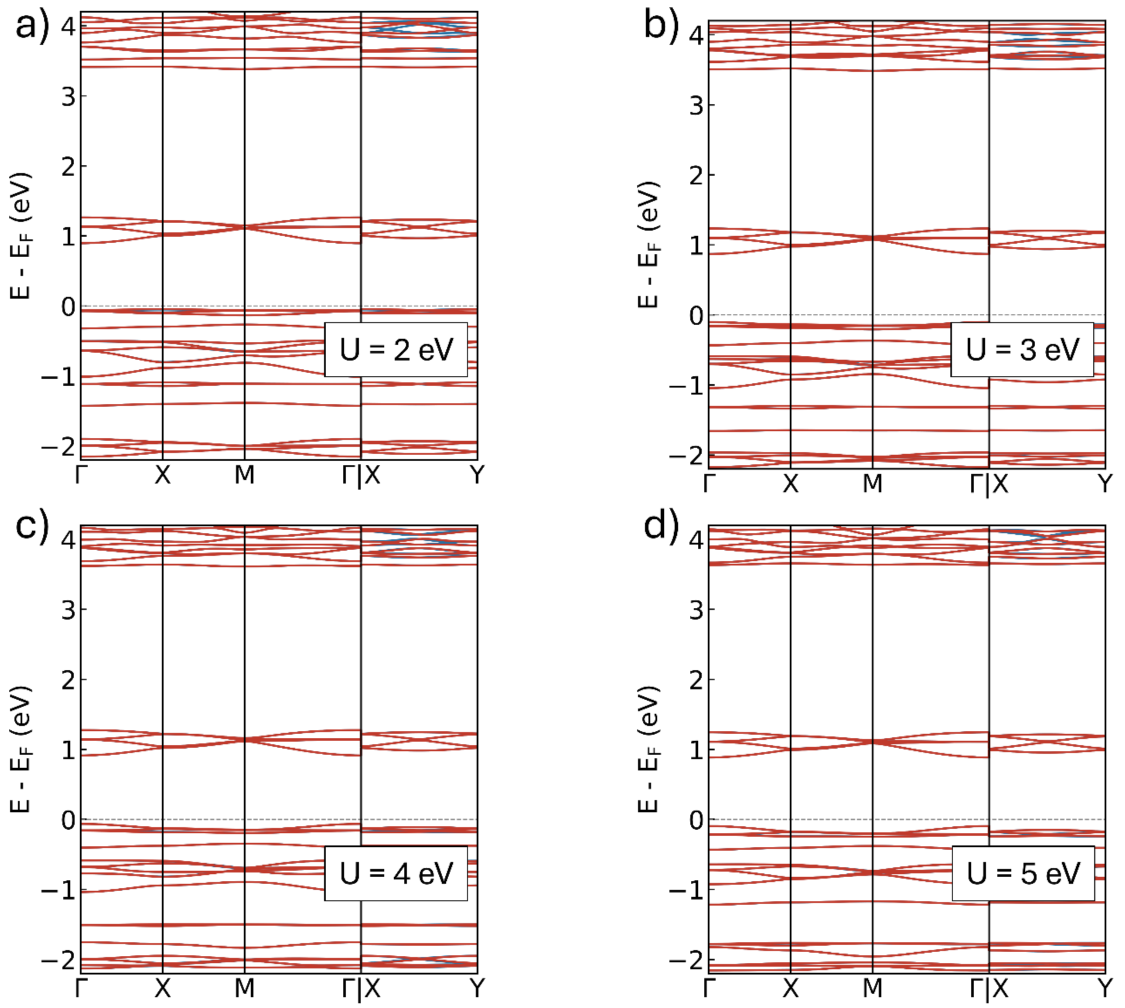


**Figure S24.** Electronic band structure of AM state in $Cr(DApent)_2$ using PBE+U. a) $U_{eff}$ = 2 eV, b) $U_{eff}$ = 3 eV, c) $U_{eff}$ = 4 eV and d) Ueff = 5 eV.

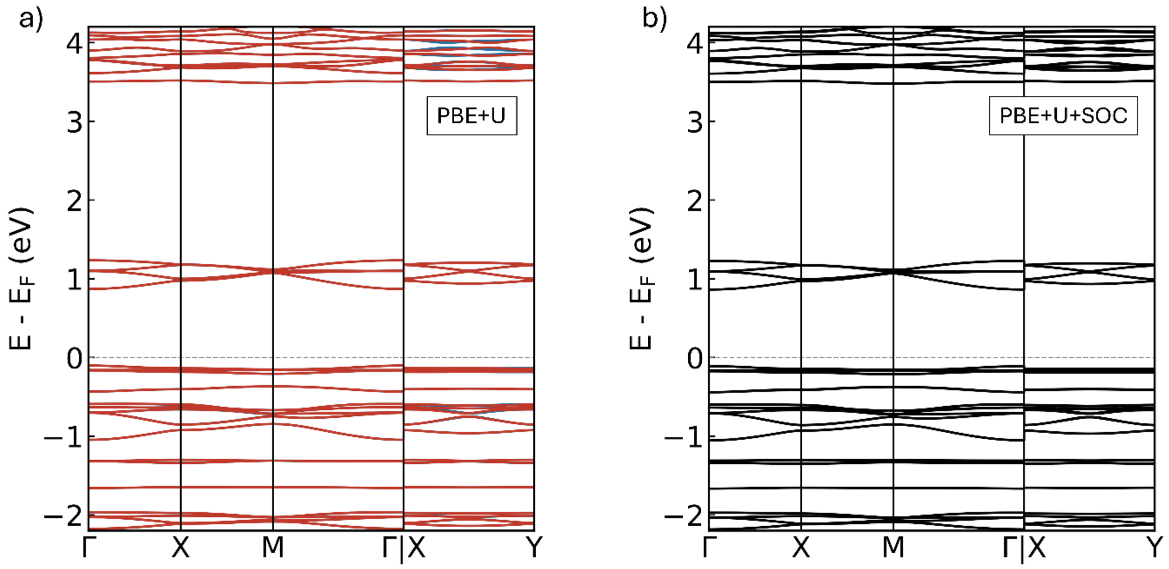


**Figure S25.** Calculated electronic band structure using PBE+U ($U_{eff}$ = 3 eV) a) without SOC and b) with SOC in AM state of $Cr(DApent)_2$.

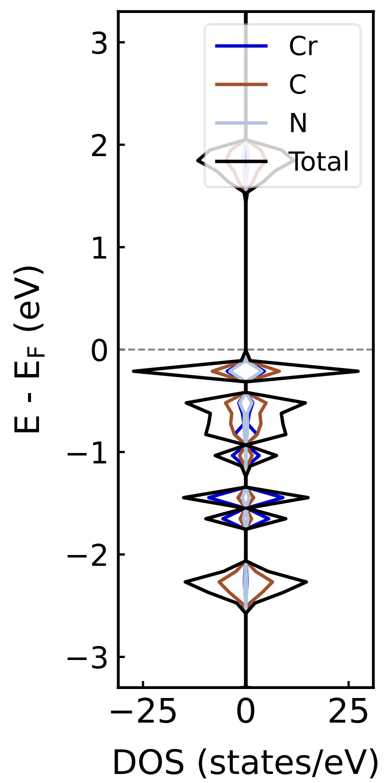


**Figure S26.** PDOS in AM state of Cr(DApent)$_2$ calculated using HSE06 functional.

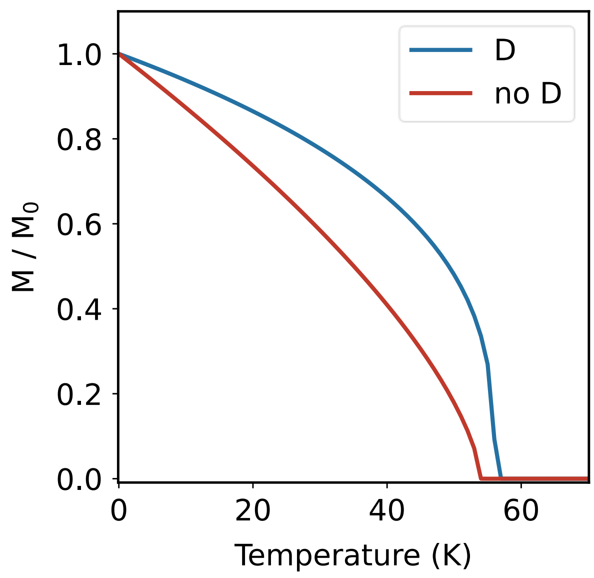


**Figure S27.** Atomistic spin dynamics simulations with (without) considering magnetic anisotropy in Cr(DApent)$_2$ in blue (red).

# 4. Cr(DAind)$_2$

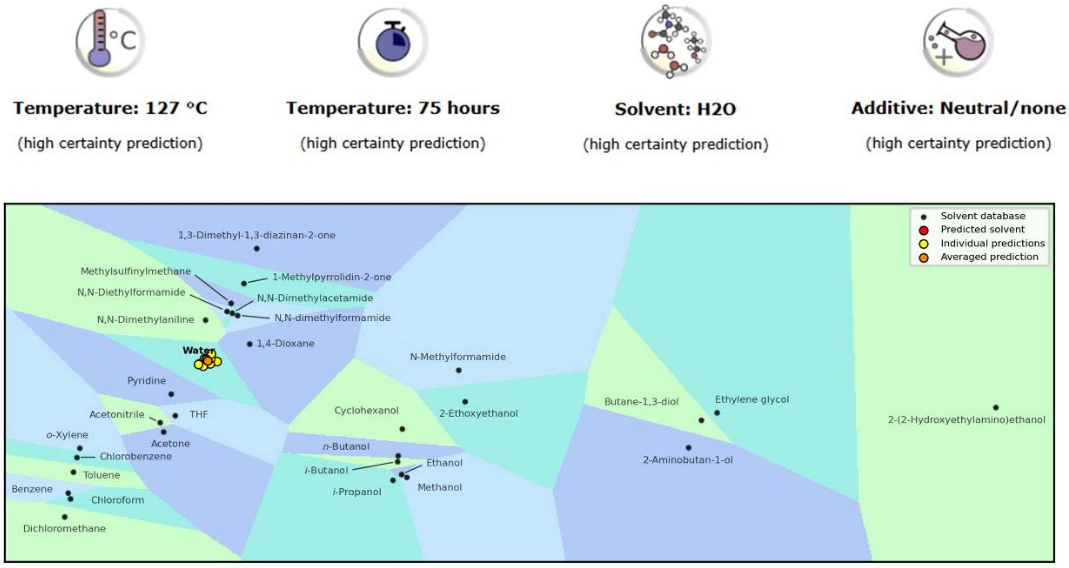


**Figure S28.** Synthesis protocol prediction of Cr(DAind)$_2$. Obtained using machine learning tool available at https://mof-synthesis.aimat.science.

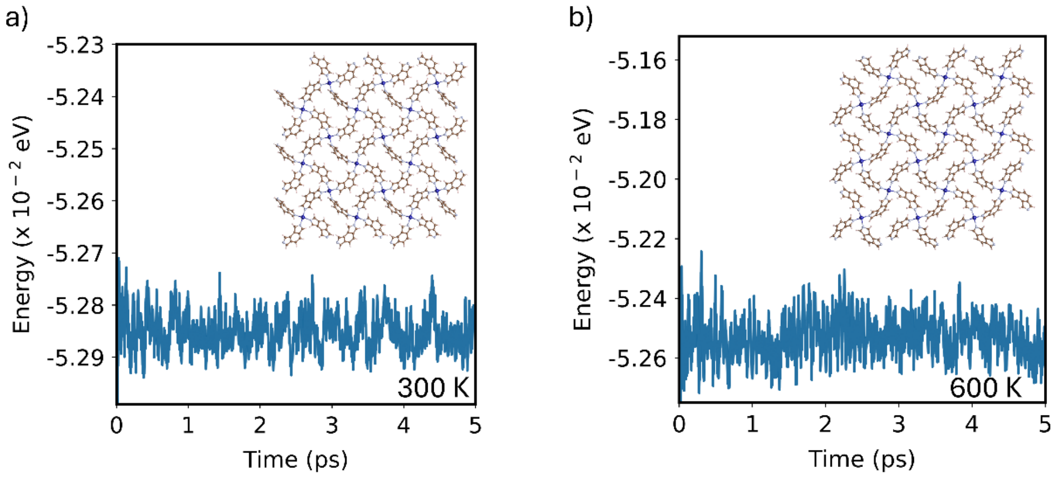


**Figure S29.** AIMD simulations at a) 300 K and b) 600 K in Cr(DAind)$_2$. Inset images represent a snapshot of the structure during the simulation.

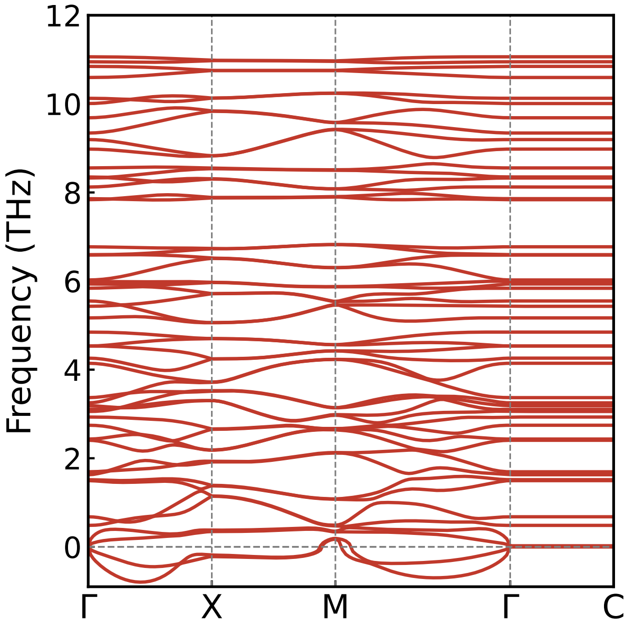


**Figure S30.** Phonon dispersion of $Cr(DAind)_2$.

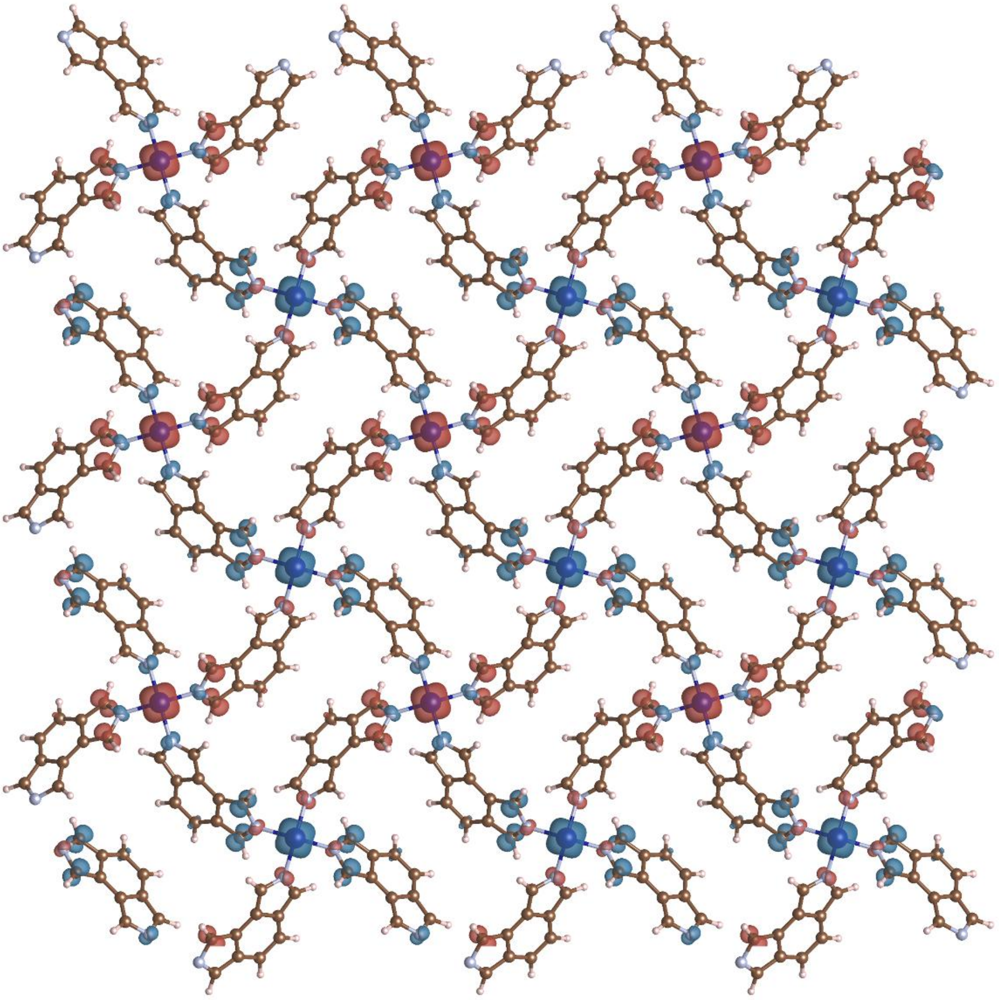

**Figure S31.** Spin density in AM state of $Cr(DAind)_2$.

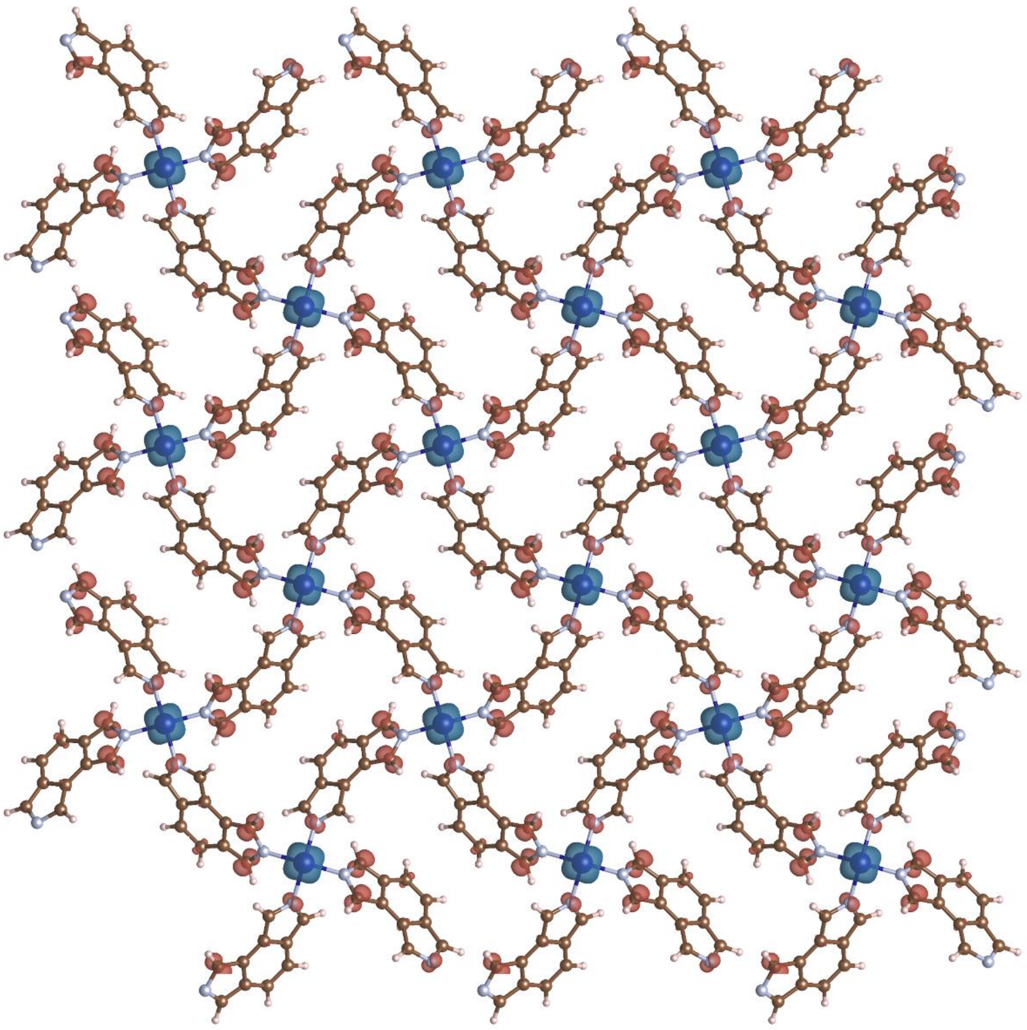

**Figure S32.** Spin density in FiM state of $Cr(DAind)_2$.

**Table S10.** Electronic band gap ($E_g$), magnetic moment in Cr atoms and ligand ($M_{Cr}$ and $M_L$ in $\mu_B$) and relative energies of different magnetic configurations FM, FiM and AM (in meV/Cr atom) in $Cr(DAind)_2$.

| | $U_{eff}$ (eV) | | | |
|---|---|---|---|---|
| | **2** | **3** | **4** | **5** |
| $\boldsymbol{E_g}$ | 0.00 | 0.00 | 0.00 | 0.00 |
| $\boldsymbol{M_{Cr}}$ | 3.53 | 3.61 | 3.69 | 3.75 |
| $\boldsymbol{M_L}$ | 0.4 | 0.4 | 0.41 | 0.41 |
| **FM** | 157.57 | 120.34 | 92.29 | 71.82 |
| **FiM** | 1.78 | 2.40 | 1.48 | 0.31 |
| **AM** | 0 | 0 | 0 | 0 |

**Table S11.** HSE06 calculated atomic magnetic moments in $Cr(DAind)_2$ unit cell.

| Atom | Magnetic moment ($\mu_B$) |
|---|---|
| Cr1 | 3.635 |
| Cr2 | -3.635 |
| C1 | -0.201 |
| C2 | 0.060 |
| C3 | -0.011 |
| C4 | -0.025 |
| C5 | 0.046 |
| C6 | 0.048 |
| C7 | 0.012 |
| C8 | 0.201 |
| C9 | -0.045 |
| C10 | 0.197 |
| C11 | -0.048 |
| C12 | -0.060 |
| C13 | 0.011 |
| C14 | 0.024 |
| C15 | -0.046 |
| C16 | -0.048 |
| C17 | -0.012 |
| C18 | 0.045 |
| C19 | -0.197 |
| C20 | 0.048 |
| C21 | -0.011 |
| C22 | -0.048 |
| C23 | -0.047 |
| C24 | 0.024 |
| C25 | 0.012 |
| C26 | -0.060 |
| C27 | 0.045 |
| C28 | -0.201 |
| C29 | 0.047 |
| C30 | -0.197 |
| C31 | 0.011 |
| C32 | 0.048 |
| C33 | 0.047 |
| C34 | -0.023 |
| C35 | -0.012 |
| C36 | 0.060 |
| C37 | -0.045 |
| C38 | 0.201 |
| C39 | -0.047 |
| C40 | 0.198 |
| N1 | -0.104 |
| N2 | 0.121 |
| N3 | 0.104 |

| | |
|---|---|
| N4 | -0.121 |
| N5 | -0.122 |
| N6 | 0.104 |
| N7 | 0.122 |
| N8 | -0.104 |
| H1 | 0.001 |
| H2 | 0.004 |
| H3 | 0.000 |
| H4 | 0.000 |
| H5 | -0.001 |
| H6 | 0.000 |
| H7 | -0.004 |
| H8 | 0.000 |
| H9 | -0.004 |
| H10 | 0.000 |
| H11 | 0.004 |
| H12 | 0.000 |
| H13 | 0.001 |
| H14 | 0.000 |
| H15 | 0.004 |
| H16 | 0.000 |
| H17 | 0.000 |
| H18 | 0.004 |
| H19 | 0.000 |
| H20 | -0.001 |
| H21 | 0.000 |
| H22 | -0.004 |
| H23 | 0.000 |
| H24 | -0.004 |
| **Total** | **0.000** |

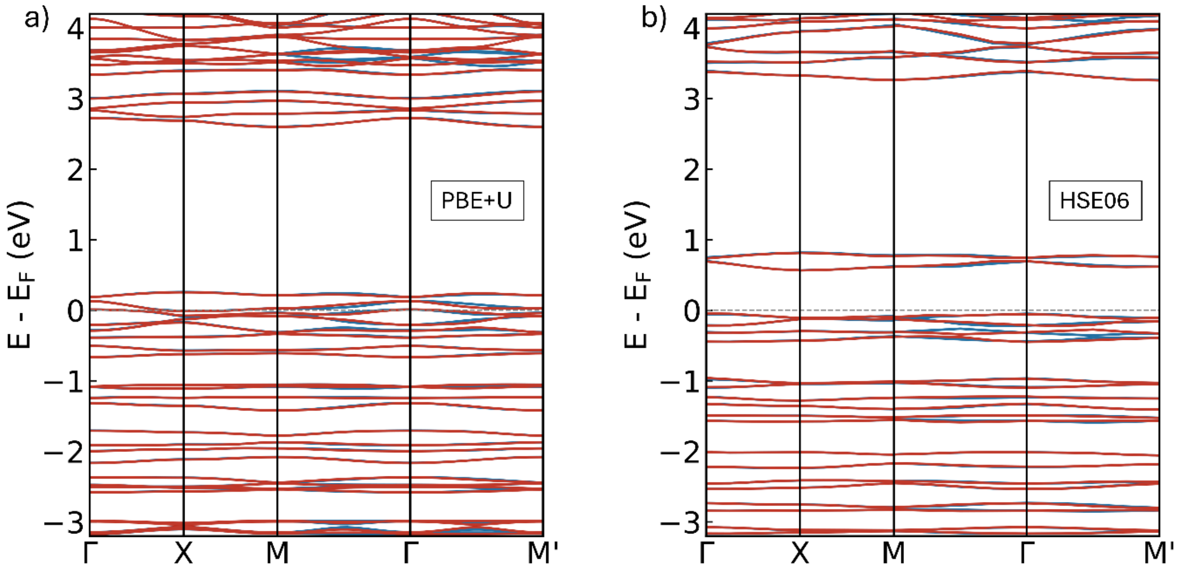


**Figure S33.** Calculated electronic band structure using a) PBE+U ($U_{eff}$ = 3 eV) and b) HSE06 functionals in AM state of $Cr(DAind)_2$.

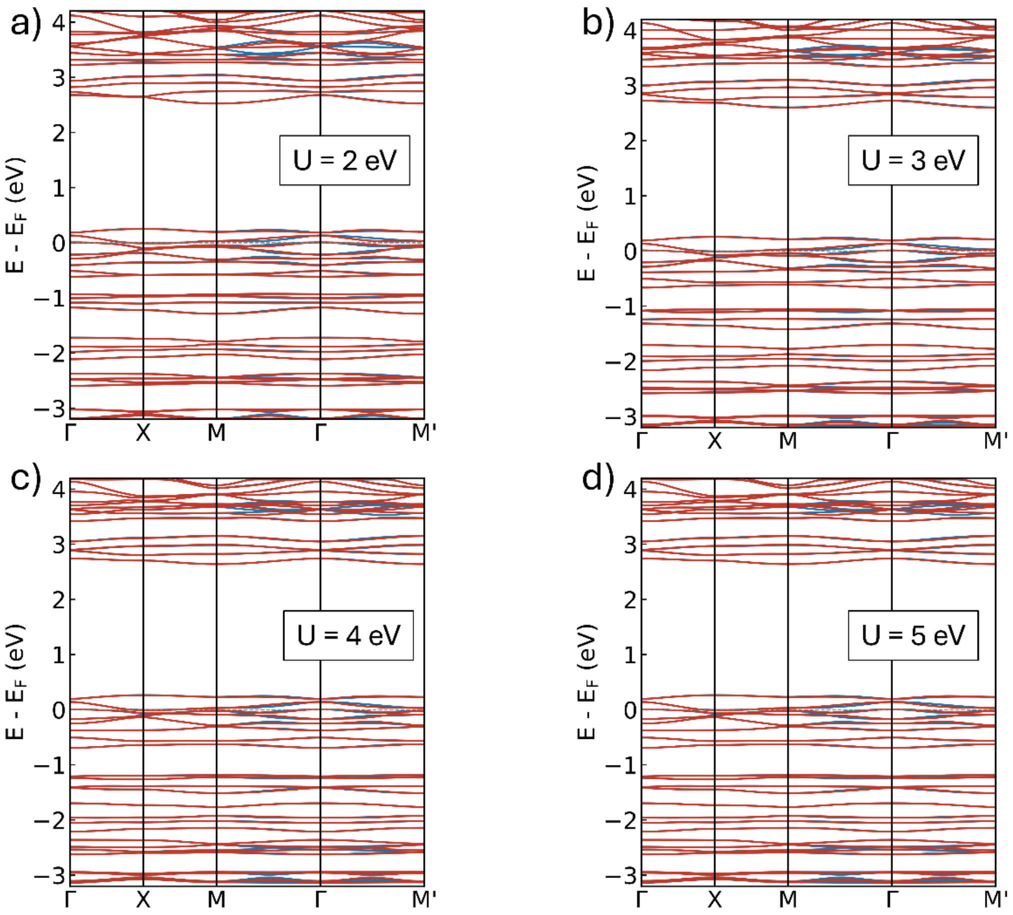


**Figure S34.** Electronic band structure of AM state in $Cr(DAind)_2$ using PBE+U. a) $U_{eff}$ = 2 eV, b) $U_{eff}$ = 3 eV, c) $U_{eff}$ = 4 eV and d) Ueff = 5 eV.

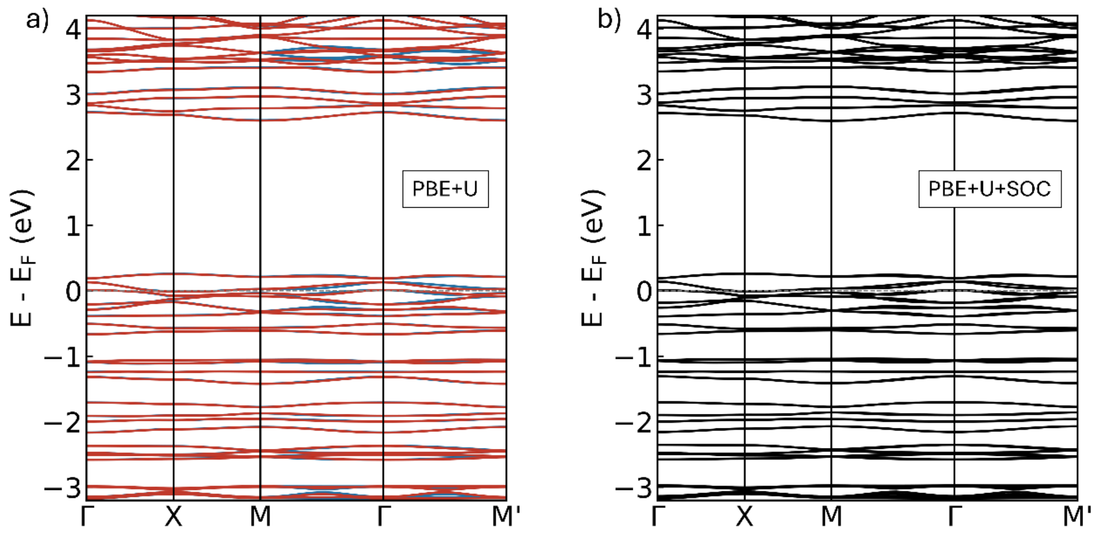


**Figure S35.** Calculated electronic band structure using PBE+U ($U_{eff}$ = 3 eV) a) without SOC and b) with SOC in $Cr(DAind)_2$.

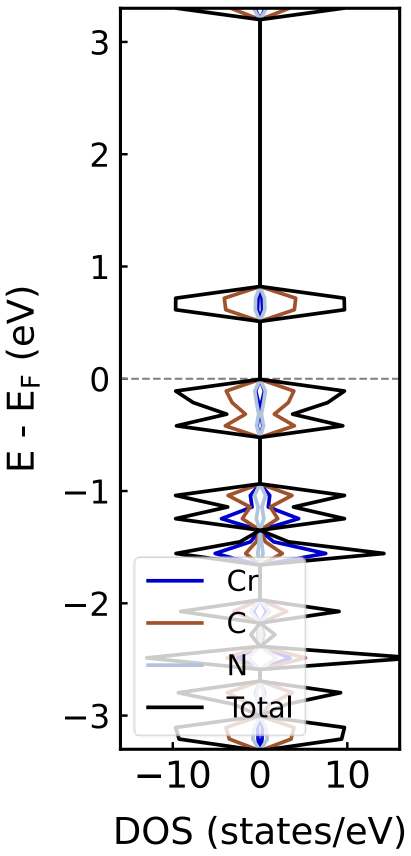


**Figure S36.** PDOS in AM state of $Cr(DAind)_2$ calculated using HSE06 functional.

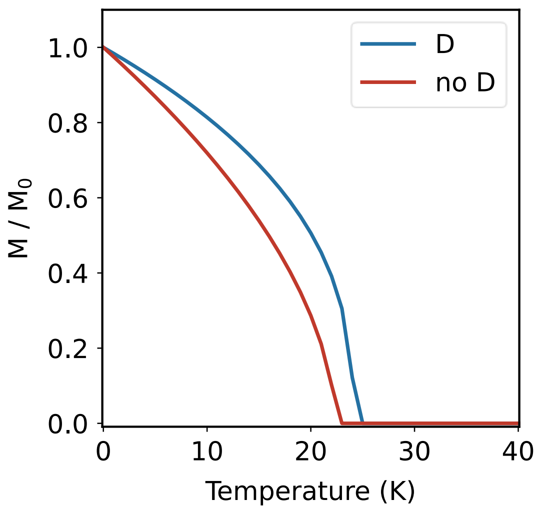


**Figure S37.** Atomistic spin dynamics simulations with (without) considering magnetic anisotropy in $Cr(DAind)_2$ in blue (red).

## 5. Symmetry analysis of $M(pyz)_2$, $Cr(imz)_2$ and $Cr(DAind)_2$

The symmetries of the $M(pyz)_2$ crystal structure are governed by the space group *P4/nbm*. In Figure S38, we illustrate the most relevant symmetry operations of this group. Because some of these symmetries require an additional spin rotation to remain valid in AFM or AM states, we describe them using non-relativistic magnetic notations. As shown in Figure S38a, the *P4/nbm* group contains a fourfold rotation about the $z$-axis, $[E \parallel C_{4z}]$, which does not involve a spin flip. The group also includes a glide symmetry $[C_2 \parallel g_z]$, consisting of a reflection in the *xy* plane followed by a translation of half a unit cell along both the *x*- and *y*-directions, as shown in Fig. S38b. This symmetry enforces trivial antiferromagnetism in $M(pyz)_2$, since it preserves the momentum direction in a two-dimensional material. Figure S38c shows four additional glide symmetries: $[C_2 \parallel g_x]$ and $[C_2 \parallel g_y]$, as well as $[C_2 \parallel g_{xy}]$ and $[C_2 \parallel g_{x\bar{y}}]$. The latter two can be obtained by combining $[E \parallel C_{4z}]$ with $[C_2 \parallel g_x]$ and $[C_2 \parallel g_y]$, respectively. The *P4/nbm* group also contains several other symmetry operations: most importantly the combination of the three gliding symmetries $g_x g_y g_z$ yields *it*-symmetry. Finally, the combinations of gliding symmetries with *it* yield 180º rotations about axes *x*- and *y-axis* without spin reversal or translations.

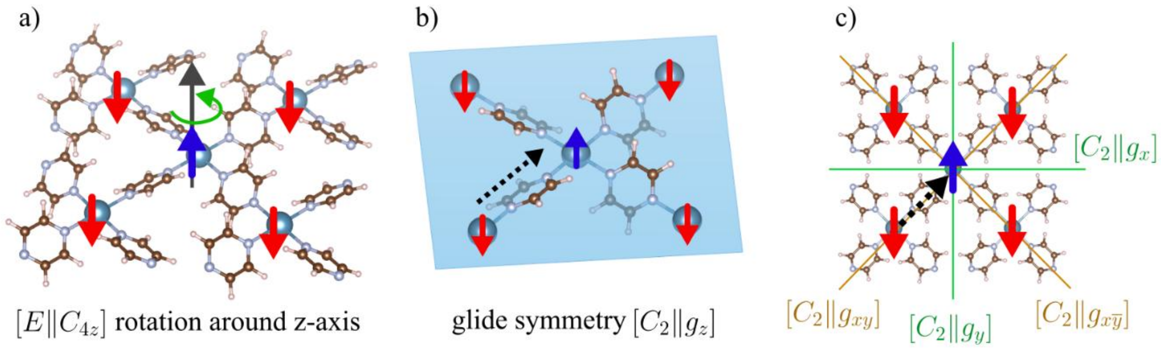


**Figure S38**. The most relevant symmetry operations of the *P4/nbm* space group: a) Fourfold (90º) rotation about the $z$-axis $[E \parallel C_{4z}]$. b) Glide symmetry $[C_2 \parallel g_z]$, (c) Glide symmetries with reflection planes perpendicular to the *xy*-plane: $[C_2 \parallel g_x]$, $[C_2 \parallel g_y]$, $[C_2 \parallel g_{xy}]$ and $[C_2 \parallel g_{x\bar{y}}]$.

The crystal symmetry is progressively reduced after the substitution of the pyz ligand with less symmetric ones. In $Cr(imz)_2$, the $[C_2 \parallel g_z]$ and $[C_2 \parallel it]$ symmetries are broken, resulting in the *P4bm* space group, as illustrated in Figure S39a. Nevertheless, the fourfold rotation $[E \parallel C_{4z}]$ and all remaining glide symmetries $[C_2 \parallel g]$ are preserved, leading to a $g$-wave altermagnetic state, as shown in Figure S39b. Although the apparent symmetry of the DAind ligand matches that of the imz ligand when considering only the atomic positions, our DFT calculations reveal a pronounced displacement in the absolute value of spin density toward one side of the ligand, thereby breaking its $C_{2v}$ symmetry (see Figure 4c in the main text). As a consequence, the space group is reduced to *Pba2* and the symmetry of the material is further lowered: both the fourfold rotation $[E \parallel C_{4z}]$ and the glide mirrors $[C_2 \parallel g_{xy}]$ and $[C_2 \parallel g_{x\bar{y}}]$ are broken. In contrast, the glide mirrors $[C_2 \parallel g_x]$ and $[C_2 \parallel g_y]$ remain intact, which forbids the emergence of ferrimagnetism. With $[E \parallel C_{4z}]$ broken, the highest remaining symmetry operation that does not require a spin flip is the twofold rotation $[E \parallel C_{2z}]$, giving rise to a $d$-wave altermagnetic state, as illustrated in Figure S39d.

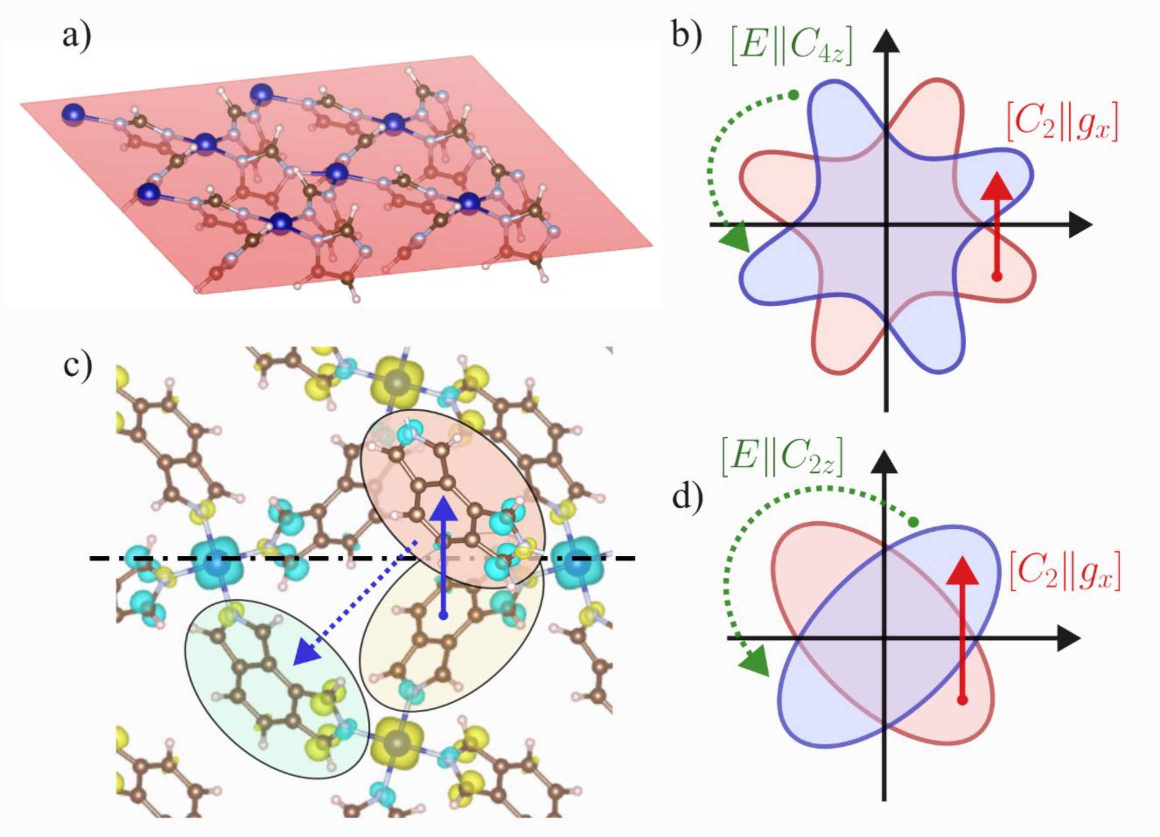


**Figure S39**. a) Illustration of the breaking of the $[C_2 \parallel g_z]$ glide symmetry in Cr(imz)$_2$, b) Combination of the $[E \parallel C_{4z}]$ rotation with the $[C_2 \parallel g_x]$ and $[C_2 \parallel g_y]$ symmetries, giving rise to g-wave altermagnetism in Cr(imz)$_2$. c) Illustration of the preservation of the $[C_2 \parallel g_x]$ glide symmetry in Cr(DAind)$_2$ despite the breaking of the intrinsic ligand symmetry. The ligand shown by the yellow ellipsoid is first reflected across the *x=0* plane (blue solid arrow, red ellipsoid) and then translated by half a unit cell along both the *x*- and *y*-directions (blue dashed arrow, green ellipsoid). When the associated spin rotation is taken into account, the transformed ligand coincides with an equivalent ligand in the crystal. d) Illustration of the $[C_2 \parallel g_x]$ and $[C_2 \parallel g_y]$ glide symmetries leading to $d$-wave altermagnetism in the absence of the $[E \parallel C_{4z}]$ rotation.

**Table S12.** List of the symmetries that are preserved in M(pyz)$_2$, Cr(imz)$_2$ and Cr(DAind)$_2$.

| | **M(pyz)$_2$** | **Cr(imz)$_2$** | **Cr(DAind)$_2$** |
|---|---|---|---|
| $[E \parallel C_{4z}]$ | yes | yes | no |
| $[E \parallel C_{2z}]$ | yes | yes | yes |
| $[C_2 \parallel g_z]$ | yes | no | no |
| $[C_2 \parallel it]$ | yes | no | no |
| $[C_2 \parallel g_{xy}]$, $[C_2 \parallel g_{x\bar{y}}]$ | yes | yes | no |
| $[C_2 \parallel g_x]$, $[C_2 \parallel g_y]$ | yes | yes | yes |

## 6. Cluster DFT Calculations

Cluster DFT calculations were performed to provide a local description of the electronic structure within the coordination environment, complementing the periodic results. To avoid ambiguity, the cluster fragments are employed here as qualitative local electronic models and are not used to assign formal charges or protonation states. This approach has been widely adopted in combined DFT studies to aid the interpretation of local electronic and magnetic properties in extended systems.[1,2]

In the cluster DFT calculations, the total charge of the $Cr(L)_4$ fragments is a technical requirement to access the physically meaningful local electronic state of the fragment. For ligands that remain electronically innocent and do not develop spin polarization in the extended framework ($Cr(imz)_4$ and $Cr(DApent)_4$), the isolated fragment naturally stabilizes in a -2 charged configuration, consistent with a closed-shell ligand environment and metal-centered spin density. In contrast, for ligands that exhibit ligand-centered spin polarization in the periodic calculations ($Cr(tdz)_4$ and $Cr(DAind)_4$), the corresponding electronic state is stabilized in a neutral fragment description, reflecting the enhanced redox activity of the ligand scaffold. Importantly, these charge states emerge as a consequence of the intrinsic electronic properties of the fragments and yield spin-density distributions fully consistent with those obtained from periodic DFT. They do not correspond to formal charge assignments or net charge differences of the MOFs, which remain globally neutral.

### 6.1 imz

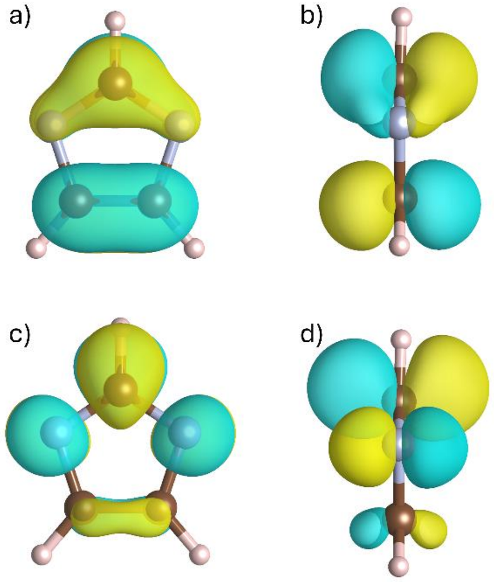


**Figure S40.** a) Top and b) side view of imz HOMO. c) Top and d) side view of imz LUMO.

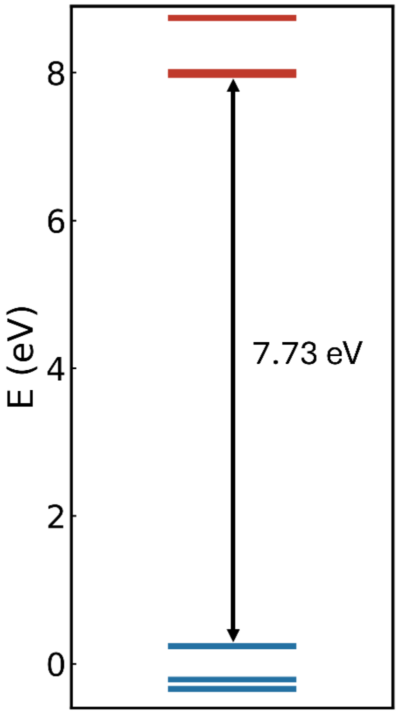


**Figure S41.** Energy diagram of frontier molecular orbitals (MO) of imz. Color code: blue (red) represents occupied (unoccupied) MOs.

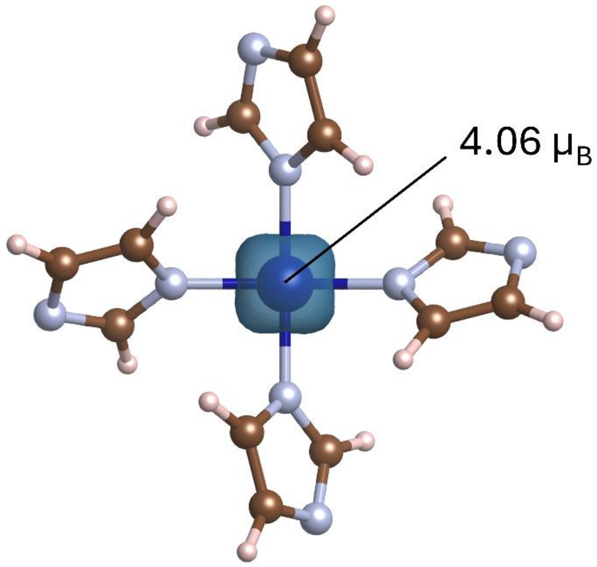


**Figure S42.** Spin density of $Cr(imz)_4$ fragment used for cluster DFT calculation. Color code: blue (red) represents spin up (down) component.

## 6.2 tdz

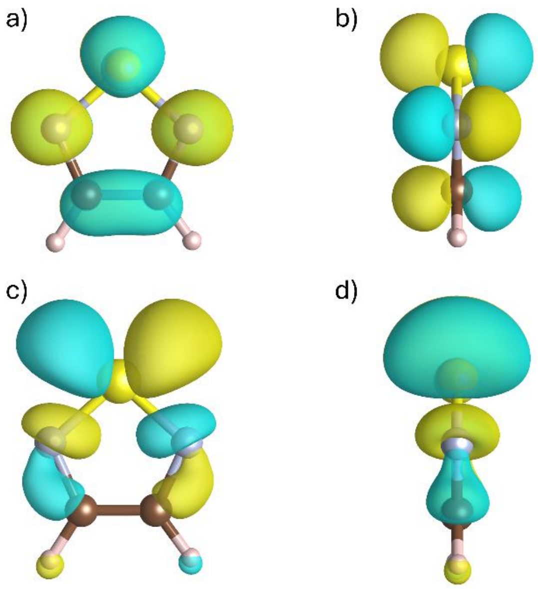


**Figure S43.** a) Top and b) side view of tdz HOMO. c) Top and d) side view of tdz LUMO.

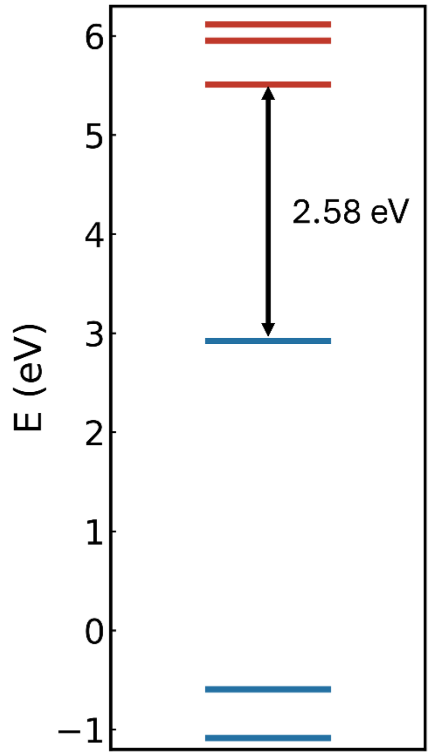


**Figure S44.** Energy diagram of frontier molecular orbitals (MO) of tdz. Color code: blue (red) represents occupied (unoccupied) MOs.

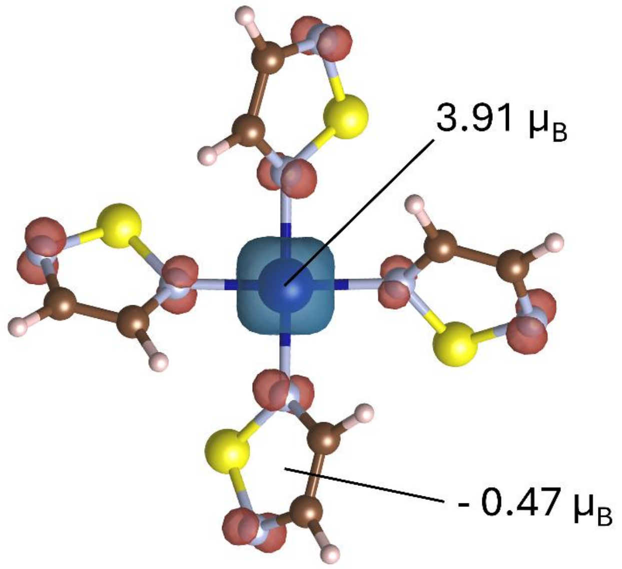


**Figure S45.** Spin density of $Cr(tdz)_4$ fragment used for cluster DFT calculation. Color code: blue (red) represents spin up (down) component.

## 6.3 DApent

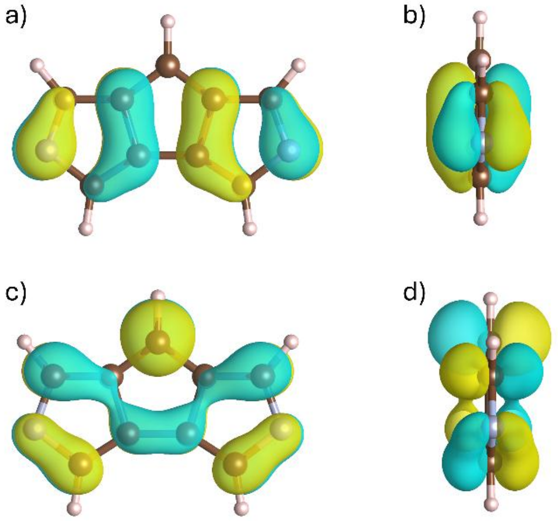


**Figure S46.** a) Top and b) side view of DApent HOMO. c) Top and d) side view of DApent LUMO.

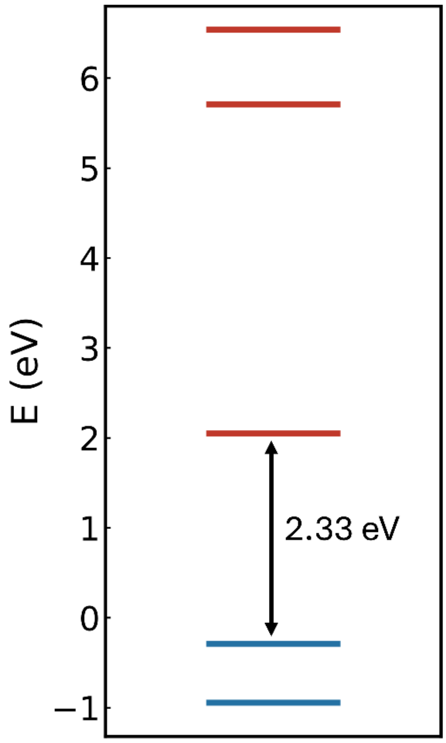


**Figure S47.** Energy diagram of frontier molecular orbitals (MO) of DApent. Color code: blue (red) represents occupied (unoccupied) MOs.

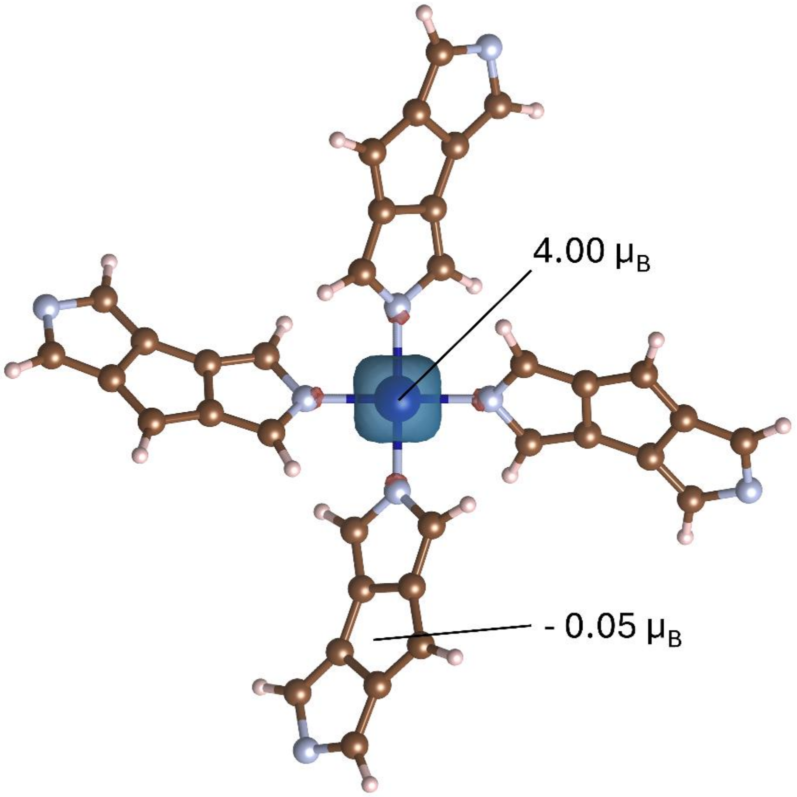


**Figure S48.** Spin density of Cr(DApent)$_4$ fragment used for cluster DFT calculation. Color code: blue (red) represents spin up (down) component.

## 6.4 DAind

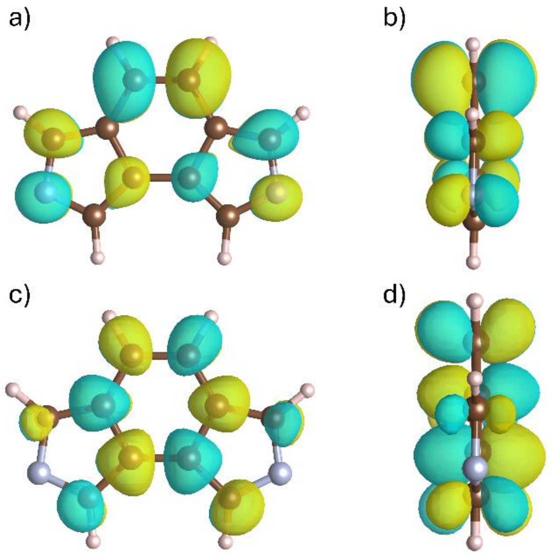


**Figure S49.** a) Top and b) side view of DAind HOMO. c) Top and d) side view of DAind LUMO.

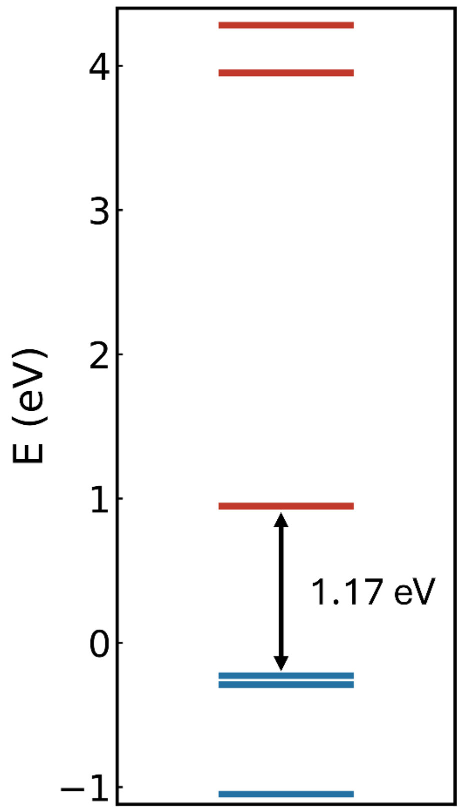


**Figure S50.** Energy diagram of frontier molecular orbitals (MO) of DAind. Color code: blue (red) represents occupied (unoccupied) MOs.

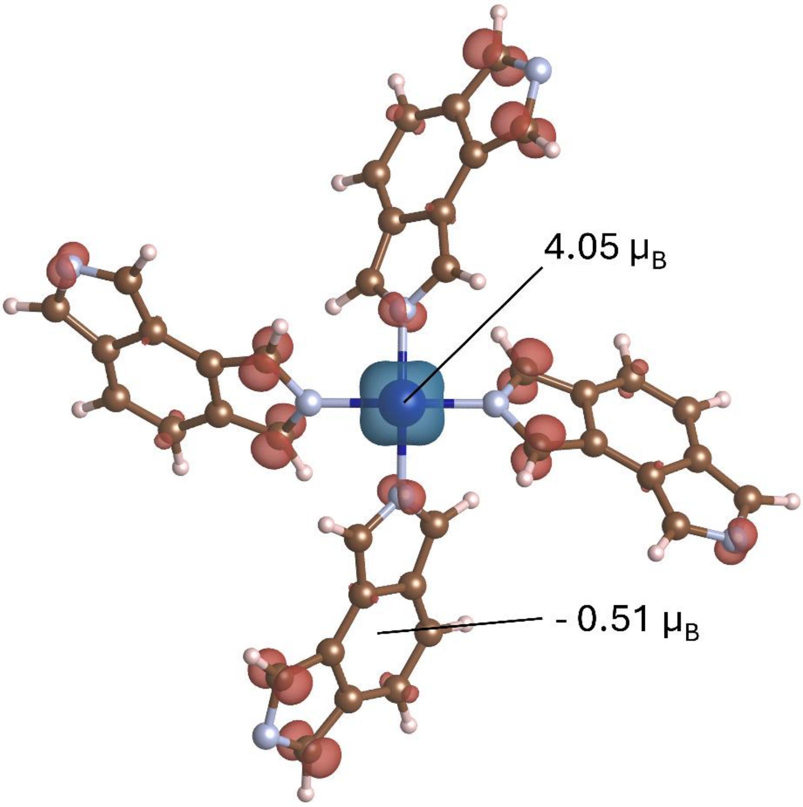


**Figure S51.** Spin density of Cr(DAind)$_4$ fragment used for cluster DFT calculation. Color code: blue (red) represents spin up (down) component.

## 6.5 Broken Symmetry Calculations

In order to support our periodic DFT calculations of metal–ligand magnetic interactions, we perform complementary cluster DFT calculations. Owing to the molecular nature of MOFs, cluster DFT benefits from a localized molecular orbital description, which does not account for periodicity but provides an additional and insightful perspective on the metal–ligand interactions. A widely used method in computational chemistry to compute magnetic exchange constants $J$ is the broken-symmetry (BS) approach proposed by Yamaguchi (Equation S1).[3,4] In this method, the energies of a high-spin state (HS) and a BS state, where the local spins are constrained to adopt antiparallel orientations, are mapped onto a Heisenberg Hamiltonian. This energy mapping provides an efficient route to estimate $J$ in systems with localized magnetic centers and has been widely applied to transition-metal complexes and coordination frameworks.

$$J = \frac{E_{BS} - E_{HS}}{\langle S_{HS} \rangle^2 - \langle S_{BS} \rangle^2} \quad (S1)$$

Therefore, we apply this methodology to $J_2$ in Cr(tdz)$_4$ and Cr(DAind)$_4$. We compute HS and BS states in Cr(tdz)$_4$ and Cr(DAind)$_4$ to evaluate $J_2$ via cluster DFT calculations. All calculations are performed in the fragments depicted in Figures S45 and S51, where we

consider $S$ = $2$ in Cr atoms and $S$ = $1$ over the ligand scaffold. Obtained results are presented in Table S13 and present a noticeable higher intensity compared to the ones reported in Table 1. We attribute this deviation mainly to the differences in magnetic moments observed between periodic and cluster DFT calculations. Here, we observe a spin polarization of 0.47 and 0.51 $\mu_B$ in Cr(tdz)$_4$ and Cr(DAind)$_4$ fragments (as depicted in Figure S45 and S51), which is double compared to the one observed in periodic DFT since there we calculate Cr(tdz)$_2$ and Cr(DAind)$_2$ due to unit cell symmetry.

**Table S13.** Relative energies of HS and BS (in meV/Cr atom) and calculated $J_2$ (in meV).

| | **HS** | **BS** | $J_2$ |
|---|---|---|---|
| **Cr(tdz)$_4$** | 572.40 | 0 | -114.18 |
| **Cr(DAind)$_4$** | 278.60 | 0 | -69.65 |

## 7. SIESTA calculations

**Table S14.** Relative energies of different magnetic configurations FM, FiM and AM (in meV/Cr atom) calculated using PBE+U ($U_{eff}$ = 3 eV) in SIESTA.

| | **Cr(imz)$_2$** | **Cr(tdz)$_2$** | **Cr(DApent)$_2$** | **Cr(DAind)$_2$** |
|---|---|---|---|---|
| **FM** | 14.66 | 1165.28 | 37.66 | 464.40 |
| **FiM** | - | 0.00 | - | 122.21 |
| **AM** | 0.00 | 391.51 | 0.00 | 0.00 |

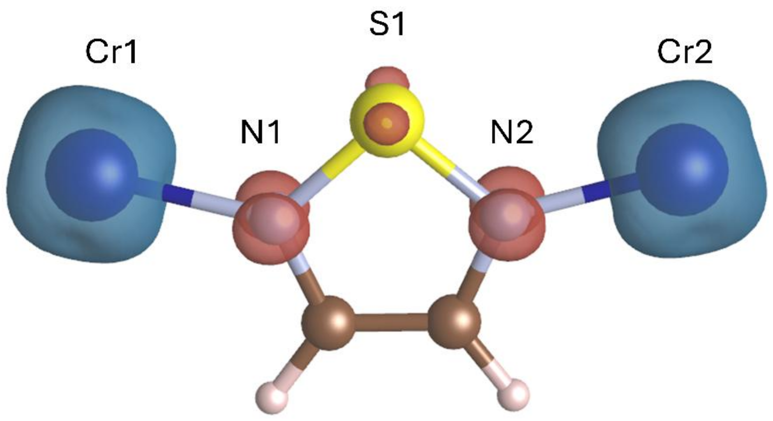


**Figure S52:** Schematic representation of Cr(tdz)$_2$ to illustrate each individual $J$. Color code: blue (red) isosurface represents spin up (down) polarization.

**Table S15.** Individual magnetic coupling between atoms belonging to tdz with corresponding metal center. Total $J_2$ corresponds to the sum of all individual magnetic couplings.

| | | ***J* (meV)** | **Distance (Å)** |
|---|---|---|---|
| **Cr1** | **N1** | -8.0958 | 4.478 |
| **Cr1** | **N1** | -0.1547 | 7.117 |
| **Cr1** | **N2** | -12.7352 | 1.982 |
| **Cr1** | **N2** | -0.1514 | 8.172 |
| **Cr1** | **S1** | -1.487 | 3.263 |
| $J_2$ = | | -22.6241 | |

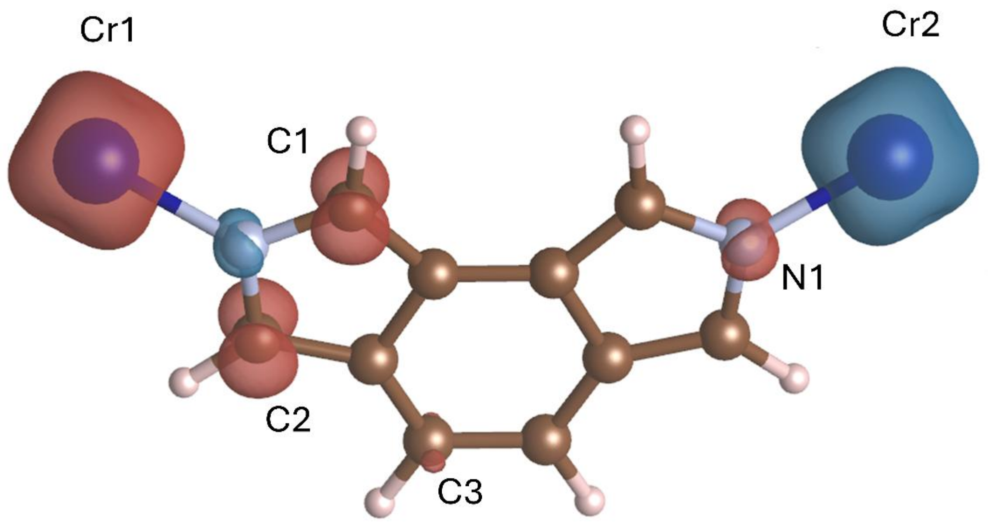


**Figure S53.** Schematic representation of Cr(DAind)$_2$ to illustrate each individual *J*. Color code: blue (red) isosurface represents spin up (down) polarization.

**Table S16.** Individual magnetic coupling between atoms belonging to DAind with corresponding metal center. Total $J_2$ corresponds to the sum of all individual magnetic couplings.

| | | ***J* (meV)** | **Distance (Å)** |
|---|---|---|---|
| **C12** | **Cr1** | 1.3165 | 3.075 |
| **C2** | **Cr1** | 2.0247 | 2.971 |
| **C3** | **Cr1** | -0.0029 | 8.124 |
| **N1** | **Cr1** | 0.0012 | 7.72 |
| | $J_2$ = | **3.3412** | |

**Table S17.** Individual magnetic coupling between atoms belonging to DAind with corresponding metal center. Total $J_2'$ corresponds to the sum of all individual magnetic couplings.

| | | ***J* (meV)** | **Distance (Å)** |
|---|---|---|---|
| **C1** | **Cr2** | -3.9839 | 6.447 |
| **C1** | **Cr2** | -0.2579 | 9.299 |
| **C2** | **Cr2** | -4.8739 | 7.796 |
| **C2** | **Cr2** | -0.8473 | 7.847 |
| **C3** | **Cr2** | -0.4906 | 6.558 |
| **N4** | **Cr2** | -2.9229 | 2.005 |
| **C3** | **Cr2** | -0.0093 | 4.187 |
| | **$J_2'$ =** | **-13.3765** | |

**Table S18.** Calculated $J$ with different $U_{eff}$ (in eV) in Cr(imz)$_2$.

| | **2** | **3** | **4** | **5** |
|---|---|---|---|---|
| **$J_1$** | -2.13 | -1.85 | -1.83 | -1.81 |

**Table S19.** Calculated $J$ with different $U_{eff}$ (in eV) in Cr(tdz)$_2$.

| | **2** | **3** | **4** | **5** |
|---|---|---|---|---|
| **$J_1$** | 2.15 | 1.72 | 1.46 | 1.23 |
| **$J_2$** | -25.67 | -22.62 | -19.63 | -17.97 |
| **$J_2'$** | -25.67 | -22.62 | -19.63 | -17.97 |

**Table S20.** Calculated $J$ with different $U_{eff}$ (in eV) in Cr(DApent)$_2$.

| | **2** | **3** | **4** | **5** |
|---|---|---|---|---|
| **$J_1$** | -3.52 | -3.20 | -2.98 | -2.73 |

**Table S21.** Calculated $J$ with different $U_{eff}$ (in eV) in Cr(DAind)$_2$.

| | **2** | **3** | **4** | **5** |
|---|---|---|---|---|
| **$J_1$** | 1.67 | 1.45 | 1.22 | 0.94 |
| **$J_2$** | 4.78 | 3.34 | 2.45 | 1.98 |
| **$J_2'$** | -15.02 | -13.37 | -11.76 | -9.11 |

## 8. Bader Charge Analysis

**Table S22.** Bader charge in *e* calculated using HSE06 hybrid DFT functional.

| | $Cr(imz)_2$ | $Cr(Dapent)_2$ | $Cr(Daind)_2$ |
|---|---|---|---|
| **Metal** | 1.56 | 1.58 | 1.58 |
| **Ligand** | -0.78 | -0.79 | -0.79 |

## 9. Linear Spin-Wave Theory Simulations

Figure S54 shows the structure of spin Hamiltonians of both g-wave and d-wave 2D AM MOFs. We have found that only a single exchange interaction $J_1$ in g-wave is strong enough to significantly affect the magnetic properties. With other exchange interactions neglected the symmetry of the spin Hamiltonian becomes higher than the symmetry of the material itself and prohibits altermagnetic splitting of the magnon modes. On the other hand, in the d-wave altermagnet the spin polarization of the ligands leads to low symmetry of the spin Hamiltonian (matching the symmetry of the material). The magnon modes calculated with the linear spin wave theory[5] display a significant splitting of the low energy modes as shown in Figure 5e of the main text. To further investigate this, we calculated the chirality of the modes following.[6] Our results show that on the ΓM branch of k-path the lowest magnon energy corresponds to the left-handed magnons (with negative chirality) while on the ΓM' branch the energy of the right-handed magnons (with positive chirality) is lower.

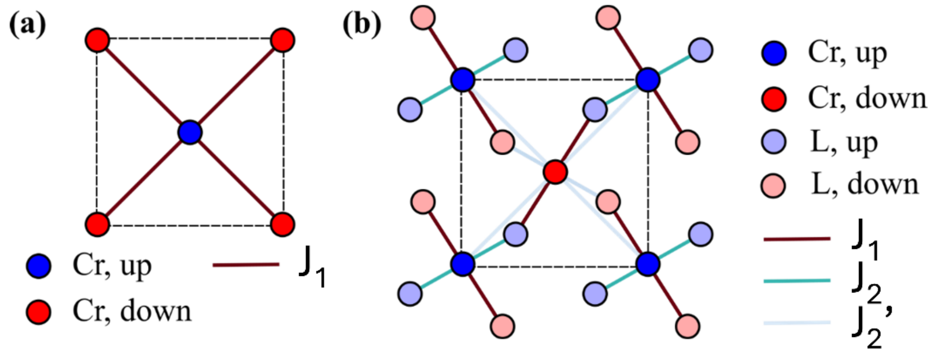


**Figure S54.** Sketch of the spin Hamiltonians of a) g-wave $Cr(imz)_2$ and b) d-wave $Cr(DAind)_2$.

## 10. Spin-Dependent Transport Calculations

To investigate spin splitting in the altermagnetic compounds $Cr(imz)_2$ and $Cr(DAind)_2$, we calculate their transport properties within the semiclassical Boltzmann formalism using a single phenomenological relaxation time $\tau$. Within this approximation, the electronic distribution function $f$ can be expanded as a series in powers of the applied electric field $\boldsymbol{E}$:

$$f_\xi^{(n)}(\boldsymbol{k}) = -\tau e \boldsymbol{E} \frac{\partial f_\xi^{(n-1)}(\boldsymbol{k})}{\hbar \partial \boldsymbol{k}} \qquad (S2)$$

Here $\xi$ labels the electron bands, and $n$ denotes the order of the non-equilibrium contribution $f^{(n)}$ with respect to the electric field. $e$ is the electron charge, $\boldsymbol{k}$ is electron wave vector, and $\hbar$ is the Plank constant. The zeroth-order contribution $f^{(0)}$ corresponds to the equilibrium Fermi-Dirac distribution.

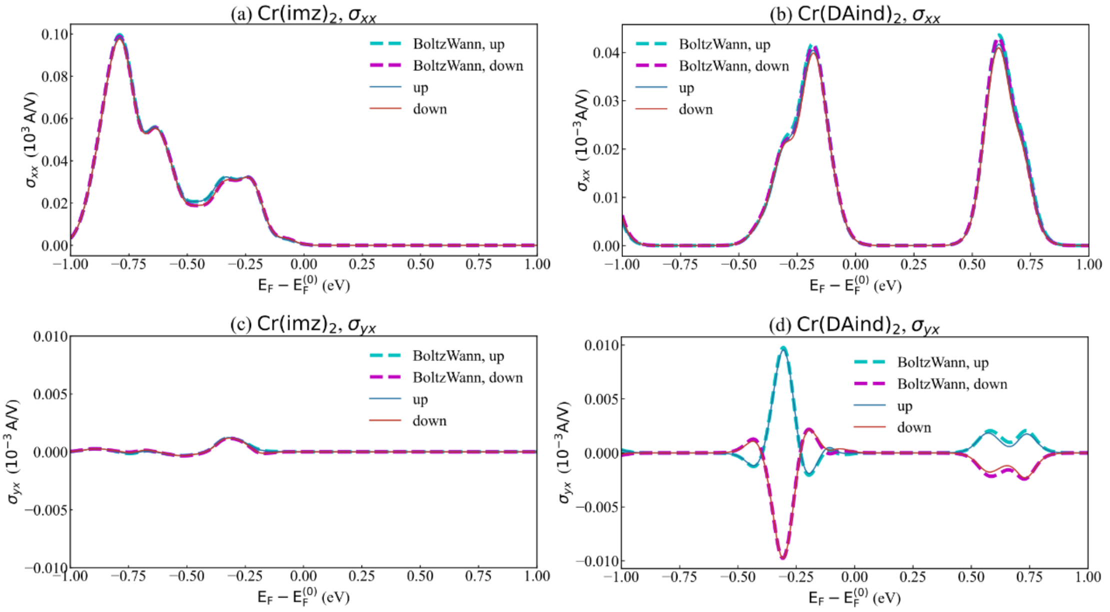


**Figure S55.** Comparison of the diagonal conductivity $\sigma_{xx}$ (a,b) and non-diagonal conductivity $\sigma_{yx}$ (c,d) calculated with Eqs. (S2) and (S3) and automatically calculated with BotzWann package. Panels a) and c) stand for g-wave Cr(imz)$_2$ and panels b) and d) for d-wave Cr(DAind)$_2$.

Because Eq. (S2) does not couple different wave vectors, it can be solved analytically and requires only the knowledge of the electron dispersion and its derivatives with respect to $\boldsymbol{k}$ up to order $n$. These quantities are obtained from tight-binding Hamiltonians constructed using the *Wannier90* code.

The contribution to the 2D current density at an arbitrary order $n$ is given by:

$$\boldsymbol{j}^{(n)} = \frac{1}{(2\pi)^2} \int d\boldsymbol{k} \sum_\xi e \boldsymbol{v} f_\xi^{(n)} \qquad (S3)$$

where $\boldsymbol{v}$ is the electron group velocity.

Up to the third order in the applied electric field, the current density can be phenomenologically expressed as

$$j_\alpha^{(s)} = \sigma_{\alpha\beta}^{(s)} E_\beta + \sigma_{\alpha\beta\gamma\delta}^{(3)(s)} E_\beta E_\gamma E_\delta \qquad (S4)$$

Here s = ↑ or ↓ labels electron spin. The tensor $\sigma_{\alpha\beta}^{(s)}$ represents the linear (*n=1*) conductivity and $\sigma_{\alpha\beta\gamma\delta}^{(3)(s)}$ describes the third order (*n=3*) response. The second-order response (*n=2*) is forbidden by symmetry in all materials considered in this work.

The conductivity tensor $\sigma_{\alpha\beta}^{(s)}$ can be calculated directly from Eqs. (S2, S3) and automatically using BoltzWann software package.[7] Figure S48 demonstrates an excellent agreement between these two approaches.

Figures 6 a) and b) in the main text show the differences between $\sigma_{yx}^{(\uparrow)}$ and $\sigma_{yx}^{(\downarrow)}$ in $Cr(imz)_2$, and between $\sigma_{yxxx}^{(3)(\uparrow)}$ and $\sigma_{yxxx}^{(3)(\downarrow)}$ in $Cr(DAind)_2$, respectively. These quantities illustrate the dependence of current-to-spin conversion on the position of the Fermi level.

Another commonly used measure of current-to-spin conversion is the spin-splitting angle $\theta$, defined as

$$\tan(\theta) = \frac{\sigma_{yx}^{(\uparrow)} - \sigma_{yx}^{(\downarrow)}}{\sigma_{xx}^{(\uparrow)} + \sigma_{xx}^{(\downarrow)}} \qquad (S5)$$

This dimensionless quantity characterizes the strength of the generated spin current relative to the conventional charge current.

To describe nonlinear spin splitting in an analogous manner, we introduce the third-order spin-splitting angle, defined as:

$$\tan\left(\theta^{(3)}\right) = \frac{\sigma_{yxxx}^{(3)(\uparrow)} - \sigma_{yxxx}^{(3)(\downarrow)}}{\sigma_{xxxx}^{(3)(\uparrow)} + \sigma_{xxxx}^{(3)(\downarrow)}} \qquad (S6)$$

Figure S56 presents the calculated spin-splitting angles $\theta^{(3)}$ and $\theta$ for $Cr(imz)_2$ and $Cr(DAind)_2$, respectively.

All calculations are performed at a temperature of 300 K with a relaxation time $\tau = 10$ fs. While it is impossible for us to predict the sample qualities in possible experiments, we can compare the selected relaxation time with the mobilities measured in other MOFs with field effect transistor (FET) and flash photolysis - time-resolved microwave conductivity (FP-TRMC) methods. They were reported to be in range from $\sim 5\times 10^{-3}$ to $\sim 100$ $cm^2V^{-1}s^{-1}$ with the highest values, probably, obtainable only in the materials of the best quality.[8,9]

While the relationship between relaxation time and the measured mobility depends on the fine properties of the band structure, and mobilities measured in different kinds of experiments can appear to be different, we use the following model for an order-of-magnitude estimate. We relate the mobility ($\mu$) to the average drift velocity of electrons in an applied electric field $\mu = \langle v_{dr}\rangle/E$. The drift velocity can be estimated from Boltzmann equation in a middle of a conductive or valence band as $\langle v_{dr}\rangle \sim \tau e E v_{gr}^2 g(\varepsilon_F) V_{uc}$, where $v_{gr}$ is a group velocity, $g(\varepsilon_F)$ – density of states at the

Fermi level, $V_{uc}$ – is the volume of a unit cell. $g(\varepsilon_F)V_{uc} \sim 1/\Delta E$ where $\Delta E$ is the width of a single band. Following this estimate and taking into account the values $v_{gr} = 4 \times 10^4$ m/s and $\Delta E = 0.1$ eV, typical for our structures, we get the mobility $\mu$ corresponding to the relaxation time of 10 fs equal to ~*1* cm$^2$V$^{-1}$s$^{-1}$. This shows that the relaxation time $\tau = 10$ fs corresponds to a typical measured mobility of a conducting MOF.

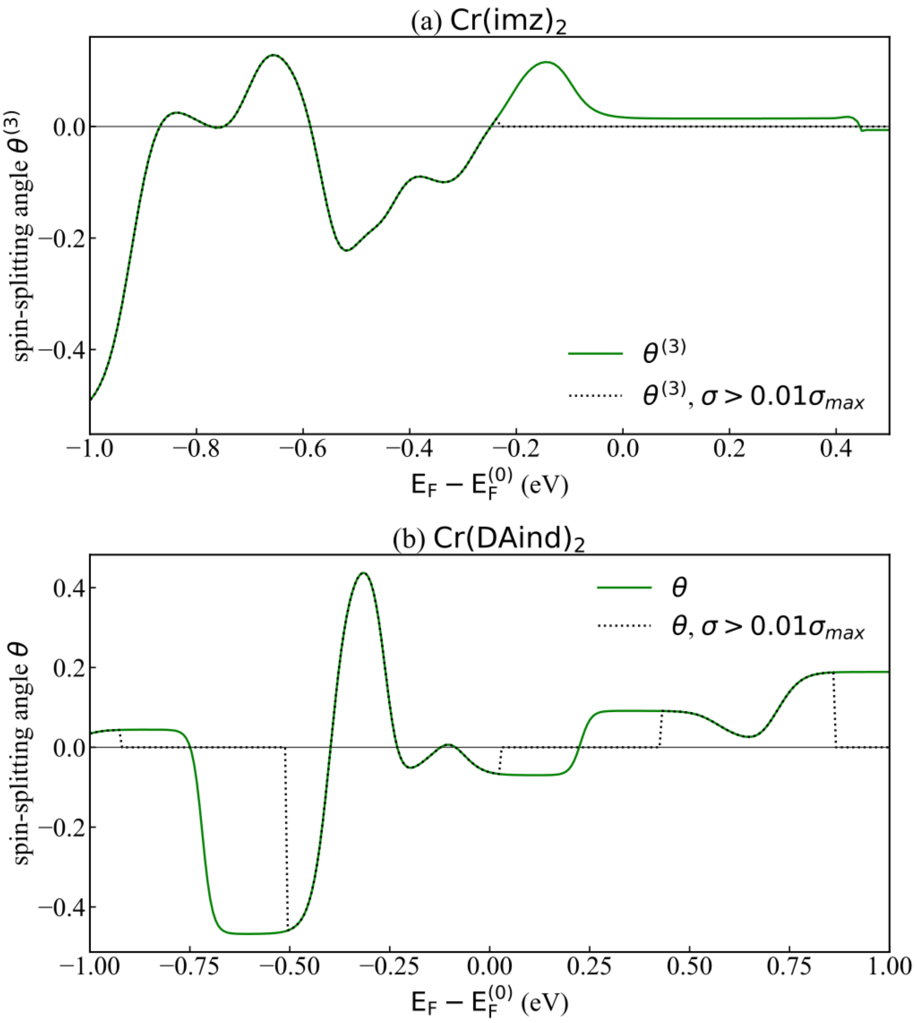


**Figure S56.** Spin splitting angles: a) $\theta^{(3)}$ calculated for g-wave Cr(imz)$_2$, b) $\theta$ calculated for d-wave Cr(DAind)$_2$.

The linear spin-splitting angle values, $\theta > 0.1$, in Cr(DAind)$_2$ indicate that this effect is sufficiently strong to be detectable in any experiment with an appropriate setup. In contrast, non-linear spin splitting is expected to become significant—or at least measurable—only at sufficiently high fields. Our calculations show that the typical linear conductivity of Cr(imz)$_2$ is on the order of ~$10^{-5}$ A/V (Figure S55 a), while the non-linear spin-splitting effect is characterized by a non-linear conductivity $\sigma_{yxxx} \sim 10^{-2}$A nm$^2$/V$^3$. This implies that under external electric fields of ~$1\ V/\mu m$ —which are moderately large

but experimentally achievable—the non-linear spin-splitting contribution would amount to roughly 0.1% of the total current, making it measurable with sufficiently sensitive techniques.

It is also noteworthy that the non-linear spin-splitting effect strongly benefits from high sample quality. For example, a tenfold increase in mobility, corresponding to a tenfold increase in relaxation time, would amplify the non-linear effect by a factor of 100 relative to the linear conductivity, potentially reaching ~10% under similar experimental conditions.

## REFERENCES


(1) Perlepe, P.; Oyarzabal, I.; Voigt, L.; Kubus, M.; Woodruff, D. N.; Reyes-Lillo, S. E.; Aubrey, M. L.; Négrier, P.; Rouzières, M.; Wilhelm, F.; Rogalev, A.; Neaton, J. B.; Long, J. R.; Mathonière, C.; Vignolle, B.; Pedersen, K. S.; Clérac, R. From an Antiferromagnetic Insulator to a Strongly Correlated Metal in Square-Lattice MCl2(Pyrazine)2 Coordination Solids. *Nat. Commun.* **2022**, *13* (1), 5766.

(2) Perlepe, P.; Oyarzabal, I.; Mailman, A.; Yquel, M.; Platunov, M.; Dovgaliuk, I.; Rouzières, M.; Négrier, P.; Mondieig, D.; Suturina, E. A.; Dourges, M.-A.; Bonhommeau, S.; Musgrave, R. A.; Pedersen, K. S.; Chernyshov, D.; Wilhelm, F.; Rogalev, A.; Mathonière, C.; Clérac, R. Metal-Organic Magnets with Large Coercivity and Ordering Temperatures up to 242°C. *Science (1979).* **2020**, *370* (6516), 587–592.

(3) Yamanaka, S.; Kawakami, T.; Nagao, H.; Yamaguchi, K. Effective Exchange Integrals for Open-Shell Species by Density Functional Methods. *Chem. Phys. Lett.* **1994**, *231* (1), 25–33.

(4) Kawakami, T.; Takeda, R.; Nishihara, S.; Saito, T.; Shoji, M.; Yamada, S.; Yamanaka, S.; Kitagawa, Y.; Okumura, M.; Yamaguchi, K. Symmetry and Broken-Symmetry in Molecular Orbital Descriptions of Unstable Molecules. 3 The Nature of Chemical Bonds of Spin Frustrated Systems. *Journal of Physical Chemistry A* **2009**, *113* (52), 15281–15297.

(5) Toth, S.; Lake, B. Linear Spin Wave Theory for Single-Q Incommensurate Magnetic Structures. *Journal of Physics: Condensed Matter* **2015**, *27* (16), 166002.

(6) Cônsoli, P. M.; Vojta, M. SU(*N*) Altermagnetism: Lattice Models, Magnon Modes, and Flavor-Split Bands. *Phys. Rev. Lett.* **2025**, *134* (19), 196701.

(7) Pizzi, G.; Volja, D.; Kozinsky, B.; Fornari, M.; Marzari, N. BoltzWann: A Code for the Evaluation of Thermoelectric and Electronic Transport Properties with a Maximally-Localized Wannier Functions Basis. *Comput. Phys. Commun.* **2014**, *185* (1), 422–429.

(8) Sun, L.; Campbell, M. G.; Dincă, M. Electrically Conductive Porous Metal–Organic Frameworks. *Angewandte Chemie International Edition* **2016**, *55* (11), 3566–3579.

(9) Xie, L. S.; Skorupskii, G.; Dincă, M. Electrically Conductive Metal–Organic Frameworks. *Chem. Rev.* **2020**, *120* (16), 8536–8580.
